# Tumors cells with mismatch repair deficiency induce hyperactivation of pyroptosis resistant to cell membrane damage but are more sensitive to co-treatment of IFN-γ and TNF-α to PANoptosis

**DOI:** 10.1038/s41420-024-01984-7

**Published:** 2024-05-13

**Authors:** Huiyan Li, Hengli Ni, Ying Li, Aijun Zhou, Xiaokang Qin, Yuqing Li, Liheng Che, Hui Mo, Chao Qin, Jianming Li

**Affiliations:** 1 Department of Pathology, Sun Yat-Sen Memorial Hospital, Sun Yat-Sen University, Guangzhou, Guangdong China; 2 Guangdong Provincial Key Laboratory of Malignant Tumor Epigenetics and Gene Regulation, Sun Yat-Sen Memorial Hospital, Sun Yat-Sen University, Guangzhou, Guangdong China

**Keywords:** Tumour immunology, Cell death and immune response

## Abstract

Hypermutated neoantigens in cancers with DNA mismatch repair deficiency (dMMR) are prerequisites for favorable clinical responses to immune-checkpoint blockade (ICB) therapy. However, TMB is not significantly associated with favorable prognosis from Preclinical and clinical studies. It implies that except for TMB, other mechanisms should be needed to contribute to successful cancer immunotherapy. We found that the hyperactivation of PANoptotic effective molecules in dMMR tumor cells caused cell membrane damage, induced ESCRT-mediated membrane repair, and protected tumor cells from the damage caused by Triton X-100, while DNA mismatch repair proficient (pMMR) tumor cells were sensitive to Triton X-100 mediating cell membrane damage due to the lack of ESCRT-mediated membrane repair. There was hyperactivation of GSDMD, GSDME, and p-MLKL in dMMR tumor cells. Co-treatment of IFN-γ and TNF-α induced rapid death of dMMR tumor cells by inducing PANoptosis including pyroptosis, apoptosis, and no necrosis. pMMR tumor cells had defects in the PANoptosis pathway and were resistant to co-treatment of IFN-γ and TNF-α. In conclusion, we can activate immune cells to release IFN-γ and TNF-α to overcome resistance to ICB treatment.

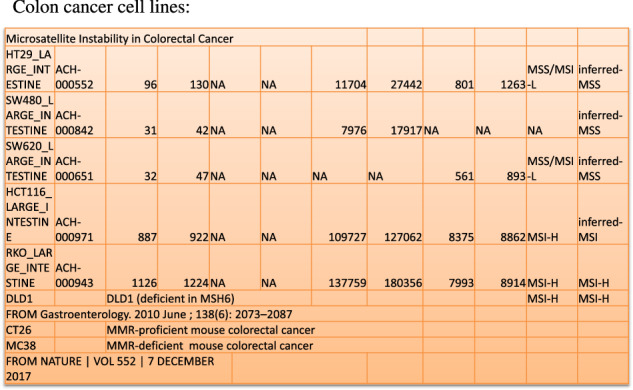

## Introduction

Cancers with DNA mismatch repair deficiency (dMMR) are prone to accumulate substantial numbers of somatic mutations, which induce high immune cell infiltration in themselves, accounting for high sensitivity to immune-checkpoint blockade (ICB) therapy [1, 2] and favorable prognosis. However, TMB is not significantly associated with favorable prognosis from preclinical and clinical studies, and the objective response rate (ORR) to anti-PD-1 therapy is quite variable, ranging from 28% to 53% [2–4]. These observations imply that except for TMB, there are other unknown mechanisms to clarify for successful cancer immunotherapy [5].

Successful cancer immunotherapy not only needs good cooperation of all kinds of immune cells but also needs cancers sensitive to killing molecules (such as proforin, granzyme, TNF-α, INF-γ) released by T cells and NK cells. Perforin can form pores in the outside plasma membrane of the target cell to let granzymes enter the cytosol and initiate PANoptosis [6]. TNF-α and IFN-γ together sensitize the cells to undergo PANoptosis without suppressing TNF-α inducing cell death by IFN-γ [7]. If cancers get a deficiency in the PANoptosis pathway, they are resistant to cancer immunotherapy treatment including ICB treatment.

Here, we show deficiency of MLH1 and subsequent accumulation of nuclear DNA activate the AIM2-ZBP-ASC-RIPK1-RIPK3-CASP8-CASP1-GSDMD-GSDME-MLKL pathway, contributing to resistance to cell membrane damage caused by Triton X-100, but more sensitive to PANoptosis induced by co-treatment of IFN-γ and TNF-α. Our findings reveal additional mechanisms of resistance to ICB therapy in dMMR cancer hosts and provide directions for future clinical practice.

## Results

### dMMR tumor cell is more sensitive than pMMR tumor cell to PANoptosis with co-treatment of IFN-γ and TNF-α

**Fig. 1:**
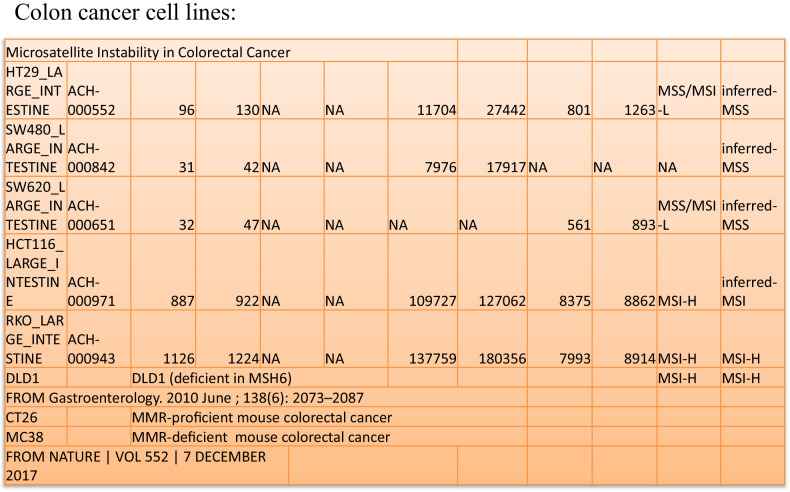
Microsatellite instability in colorectal cancer cell lines. All cells were treated by BMCyclin (Roche) for elimination of mycoplasma, and detected by MycoBlue Mycoplasma Detector kit (Vazyme company).

**Fig. 2:**
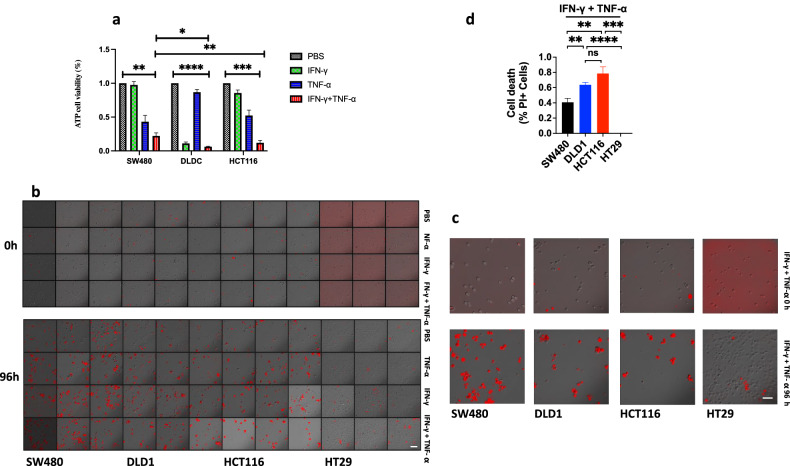
Treatment of IFN-γ, TNF-α, or IFN-γ + TNF-α induces PANoptosis. SW480, DLD1, HCT116, or HT29 were treated by PBS, IFN-γ, TNF-α, or IFN-γ + TNF-α for 96 h. Cell death was measured by ATP-based cell viability (**a**) and PI staining (**b**, **c**). Data are expressed as mean ± s.d. from three technical replicates. (mean ± s.e.m.). Two-tailed unpaired Student’s *t*-test was used to determine significance (*P < 0.05; **P < 0.01; ***P < 0.001; ****P < 0.0001; NS, not significant). Scale bars, 50 um.

We induced dMMR tumor cell lines DLD1 or HCT116 to PANoptosis with IFN-γ, TNF-α, or co-treatment of IFN-γ and TNF-α compared with pMMR tumor cell lines SW480 or HT29 (Fig. 1). The ATP release result showed IFN-γ induced little death in SW480 and HCT116, but much in DLD1; TNF-α could induce more cell death than IFN-γ in SW480 and HCT116, but little death in DLD1 (Fig. 2a); the ATP release and PI staining results showed co-treatment of IFN-γ and TNF-α induced most cell death in DLD1, HCT116, SW480, and HT29, the number of death cells in DLD1 and HCT116 was more than that in SW480 and HT29, and DLD1, HCT116 are dMMR cancer cell lines, SW480 and HT29 are pMMR cancer cell lines. The result suggested that dMMR tumor cells were more sensitive than pMMR tumor cells to PANoptosis with co-treatment of IFN-γ and TNF-α (Fig. 2a–d).

### Co-treatment of IFN-γ and TNF-α induces PANoptosis of SA-b-gal-positive (blue) cell

**Fig. 3:**
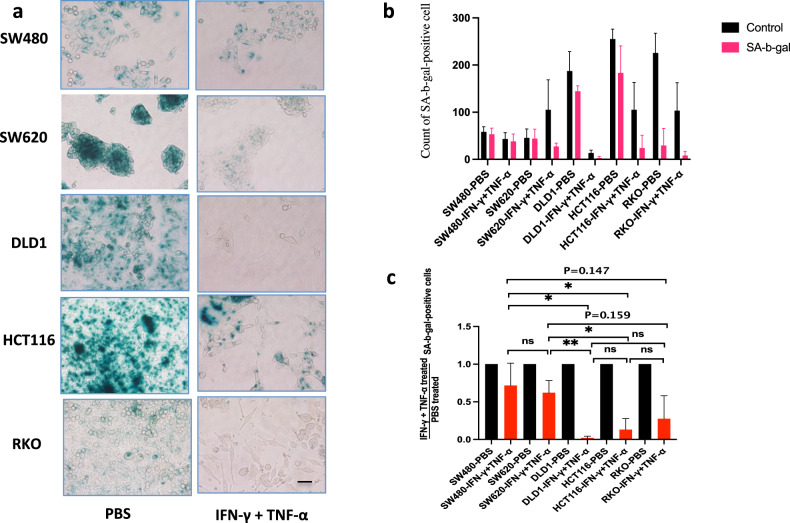
Co-treatment of IFN-γ and TNF-α induces PANoptosis of SA-b-gal-positive (blue) cells. The PANoptosis of SA-b-gal-positive (blue) cells in SW480, SW620, DLD1, HCT116, or RKO was induced by PBS or IFN-γ + TNF-α (**a**–**c**). SA-b-gal, SA-b-gal-positive (blue) cells. Data are expressed as mean ± s.d. from three technical replicates. (mean ± s.e.m.). Two-tailed unpaired Student’s *t*-test was used to determine significance (*P < 0.05; **P < 0.01; NS, not significant). Scale bars, 50 µm.

Co-treatment of IFN-γ and TNF-α can induce senescence in cells, we analyzed whether senescent cells were sensitive to co-treatment of IFN-γ and TNF-α to induce cell death [8–10]. After co-treatment of IFN-γ and TNF-α, SA-b-gal-positive (blue) cells reduced, it showed co-treatment of IFN-γ and TNF-α would induce PANoptosis of SA-b-gal-positive (blue) cells (Fig. 3a), the death rate of SA-b-gal-positive (blue) cells was positive relation with sensitivity to co-treatment of IFN-γ and TNF-α. The number of death cells in SA-b-gal-positive DLD1, HCT116, or RKO was more than that in SA-b-gal-positive SW480 or SW620, and DLD1, HCT116 and RKO are dMMR cancer cell lines, SW480 and SW620 are pMMR cancer cell lines (Fig. 1), it showed that SA-b-gal-positive dMMR cancer cell lines were more sensitive than SA-b-gal-positive pMMR cancer cell lines to co-treatment of IFN-γ and TNF-α (Fig. 3b, c). It suggested that the signal path in senescence was crossed with that in PANoptosis.

### Effect of pan-caspase inhibitor on PANoptosis with co-treatment of IFN-γ and TNF-α

**Fig. 4:**
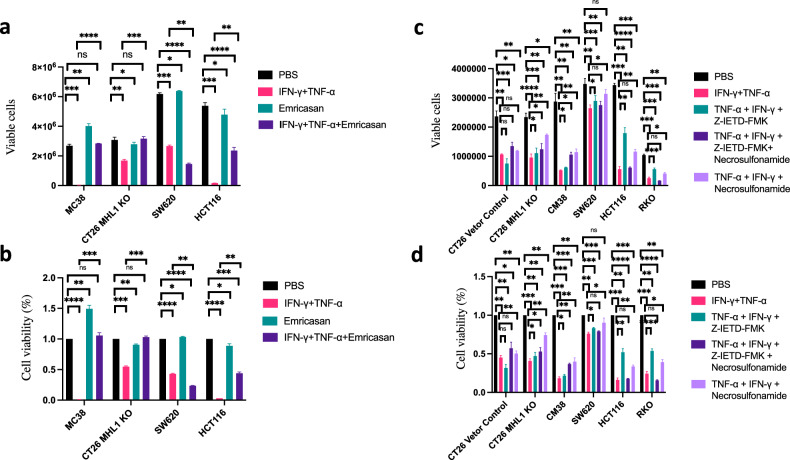
Co-treatment of TNF-α, IFN-γ, Emricasan (IDN-6556), Z-IETD-FMK, and Necrosulfonamide induces PANoptosis. MC38, CT26 MLH1 KO, SW620, HCT116 were treated with PBS, IFN-γ + TNF-α, Emricasan, or IFN-γ + TNF-α + Emricasan (**a**, **b**); PBS, IFN-γ + TNF-α, IFN-γ + TNF-α + Z-IETD-FMK, IFN-γ + TNF-α + Z-IETD-FMK +Necrosulfonamide, IFN-γ + TNF-α + Necrosulfonamide to induce PANoptosis (**c**, **d**). Viable cells were counted by Countstar Automated Cell Counter, % of Viable cells were shown. Data are expressed as mean ± s.d. from three technical replicates. (mean ± s.e.m.). Two-tailed unpaired Student’s *t*-test was used to determine significance (*P < 0.05; **P < 0.01; ***P < 0.001; ****P < 0.0001; NS, not significant).

We further analyzed the effect of caspase inhibitors on PANoptosis induced by co-treatment of IFN-γ and TNF-α. The results showed that most cancer cell line death was induced by co-treatment of IFN-γ and TNF-α, in which cell death was significantly induced in MC38 and HCT116, they were close to complete death after co-treatment of IFN-γ and TNF-α for 96 h. The pan-caspase inhibitor Emricasan alone would not inhibit cell death, along with the same growth rate of cells compared with the group treated with PBS, but it could promote cell proliferation in MC38; Emricasan suppressed cell death induced by co-treatment of IFN-γ and TNF-α, especially completely inhibiting cell death in MC38 or *Mlh1* KO CT26 co-treated with IFN-γ and TNF-α. Emricasan also inhibited cell death in HCT116 with co-treatment of IFN-γ and TNF-α, especially inhibiting 50% of cell death. During the experiment, we observed that emricasan also completely inhibited cell death in HCT116 after co-treatment of IFN-γ and TNF-α, but the cell activity was only 50% of that of control group with PBS, which was due to inducing dormancy in HCT116 after co-treatment of IFN-γ plus TNF-α plus Emricasan; Emricasan could not suppress cell death in SW620 with co-treatment of IFN-γ and TNF-α, which also explained that in addition to inducing caspase activation and cell death by co-treatment of IFN-γ and TNF-α, there were other factors to induce cell death such as inducing necrosis or activating p-H2A to induce genome damage [11] (Fig. 4a, b). We further analyzed the effects of other inhibitors on cell death. After co-treatment of IFN-γ and TNF-α for 96 h, a large number of cell death was induced, and the caspase-8 inhibitor Z-IETD-FMK could partially prevent cell death in MC38, *Mlh1* KO CT26, SW620, or HCT116, especially inhibiting 50% of cell death in HCT116 and RKO, indicating that caspase-8 plays an important role on cell death in HCT116 and RKO induced by co-treatment of IFN-γ and TNF-α, but Z-IETD-FMK could not inhibit the cell death of vector control CT26 induced by co-treatment of IFN-γ and TNF-α; Necrosulfonamide could superimpose on the inhibition of Z-IETD-FMK on cell death induced by co-treatment of IFN-γ and TNF-α in MC38, vector control CT26, and *Mlh1* KO CT26, but not in SW620, HCT116, and RKO. MLKL inhibitor Necrosulfonamide could inhibit cell death induced by co-treatment of IFN-γ and TNF-α in MC38, vector control CT26, *Mlh1* KO CT26, SW620, HCT116, or RKO, among which the inhibition was the most significant in SW620, indicating that the necrosis pathway plays an important role on cell death of SW620 induced by co-treatment of IFN-γ and TNF-α (Fig. 4c, d).

### Caspase inhibitor Emricasan can inhibit the PANoptosis induced by co-treatment of IFN-γ and TNF-α

**Fig. 5:**
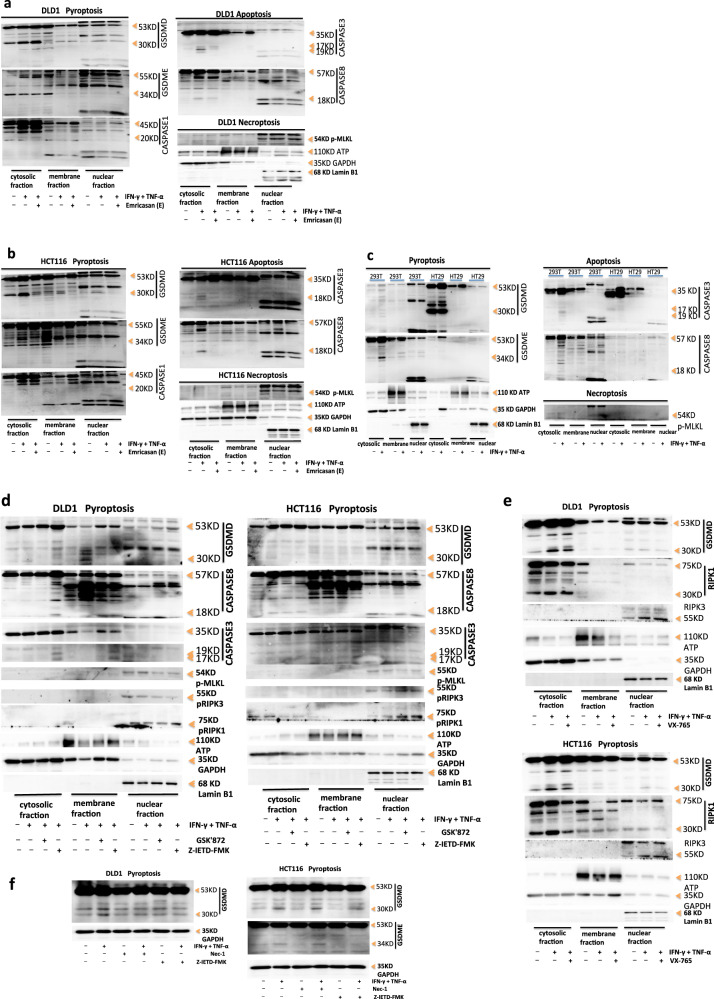
The effect of inhibitors on PANoptosis induced by co-treatment of IFN-γ and TNF-α. Immunoblot analysis of pro-(P53), activated (P30) GSDMD, pro-(P53) and activated (P34) GSDME, pro-(P45) and activated (P20) CASP1; pro-(P35) and cleaved (P19 and P17) CASP3, pro-(P55) and cleaved (P18) CASP8, phosphorylated MLKL (pMLKL), phosphorylated RIPK1 (pRIPK1), and pro-(P75) and cleaved (P30) RIPK1, phosphorylated RIPK3 (pRIPK3), RIPK3 in DLD1, HCT116 (**a**, **b**, **d**–**f**), 293 T or HT29 (**c**) after co-treatment with IFN-γ and TNF-α or supplement with Emricasan, GSK'872, Z-IETD-FMK, VX-765 or Nec-1 for 24 h. ATP, GAPDH, and Lamin B1 were used as membrane, cytosolic, and nuclear internal control respectively. Data are representative of at least three independent experiments.

We next identified specific targeting of these molecules in PANoptosis induced by co-treatment with IFN-γ plus TNF-α plus Emricasan. We further investigated the localization of death complexes within cells. Under PBS treatment, DLD1 GSDMD and HCT116 GSDMD, GSDME could also be activated and cleaved. The GSDMD-activated fragment P30 and GSDME-activated fragment P34 were naturally hyperactivated, but there were no activated fragments in the cell membrane, so there were no polymers forming on the cell membrane, no forming pores, and no inducing pyrosis (Fig. 5a, b). DLD1 did not express GSDME. DLD1 caspase-8 exhibited natural hyperactivation. Caspase-8 and caspase-1 in the cytoplasm were activated by dsDNA-AIM2-ZBP1-ASC-RIPK3-RIPK1-capase-8-caspase-1 in the nucleus, GSDMD was cleaved by activated caspase-8 and caspase-1, and caspase-3 was cleaved by activated caspase-8. IFN-γ and TNF-α co-treatment enhanced the activation of caspase-8, caspase-1, and caspase-3 in the cytoplasm, and ultimately enhanced the activation of GSDMD in the cytoplasm. Pan-caspase and caspase-8 inhibitors could inhibit the activation of caspase-3 and some caspase-8, but could not inhibit the activation of caspase-1, which was due to the activation of ASC-Caspase-8-Caspase-1 inflammasome in the nucleus, leading to sustained activation of caspase-1, caspase-8, and GSDMD, which was not affected by caspase inhibitors. Caspase-8 inhibitor could inhibit the activation of GSDMD, which’s activity was not affected by p-RIPK1 activity (Fig. 5a, d, f). HCT116 treated with PBS phosphorylated RIPK1 in the nucleus through nuclear dsDNA-AIM2-ZBP1-ASC-RIPK3-RIPK1, p-RIPK1 transported to the cytoplasm. p-RIPK1 in the cytoplasm activated caspase-8 in the cytoplasm, activated caspase-8 cleaved caspase-1 and caspase-3. Then, activated caspase-8 and caspase-1 cleaved GSDMD, and activated caspase-3 cleaved GSDME, which was regulated by p-RIPK1 activity. IFN-γ and TNF-α co-treatment enhanced the activation of caspase-8, caspase-1, and caspase-3 in the cytoplasm in HCT116, and ultimately enhanced the activation of GSDMD and GSDME. Pan-caspase and caspase-8 inhibitors could inhibit natural hyperactivation and IFN-γ plus TNF-α enhancing-activation of caspase-8, caspase-3, GSDMD, and GSDME, as well as partial activation of caspase-1. RIPK1 inhibitor could only inhibit natural hyperactivation of GSDMD and GSDME, but could not inhibite IFN-γ plus TNF-α enhancing-activation of GSDMD and GSDME. And GSDMD activity was mainly regulated by caspase-8 (Fig. 5b, d, f). RIPK3 was located in the nucleus in DLD1 or HCT116, and IFN-γ and TNF-α co-treatment enhanced expression of RIPK3. Both PBS and IFN-γ plus TNF-α could induce phosphorylation of RIPK3, which was mainly located in the nucleus in DLD1 or HCT116. RIPK1 was expressed in the cytoplasm, cell membrane, and nucleus in DLD1 or HCT116. Both PBS and IFN-γ plus TNF-α could phosphorylate RIPK1, p-RIPK1 was located in the nucleus in DLD1, but located in the cytoplasm and nucleus in HCT116; caspase-8 inhibitor enhanced expression of p-RIPK1 in the cytoplasm and nucleus in DLD1, but reduced expression of p-RIPK1 in the cytoplasm and enhanced its expression in the nucleus in HCT116 (Fig. 5d, e). p-MLKL was induced in DLD1 or HCT116 with treatment of PBS, IFN-γ and TNF-α or Emricasan plus IFN-γ plus TNF-α, but p-MLKL was only activated in the nucleus, and there were no differences in activation level among the treatment of PBS, IFN-γ and TNF-α or Emricasan plus IFN-γ plus TNF-α. Emricasan could not inhibit p-MLKL activation in DLD1 or HCT116, indicating that p-MLKL was not activated through the caspase pathway. In addition, there was no activated p-MLKL in the cytoplasm or membrane in DLD1 or HCT116, indicating that IFN-γ and TNF-α could not induce cell death by necrosis (Fig. 5a, b, d). IFN-γ and TNF-α induced PANoptosis by pyrosis and apoptosis, but not by necrosis. Emricasan treatment could inhibit pyrosis and apoptosis but had no effect on necrosis to induce PANoptosis.

We further identified specific molecules of PANoptosis in 293T or HT29 with co-treatment of IFN-γ and TNF-α. It showed that GSDMD was not expressed in 293T. GSDMD in HT29 could also be activated and cleaved by PBS treatment. The activated fragment P30 was naturally hyperactivated, but there was no activated fragment P30 in the cell membrane, so it did not induce pyrosis. 100 ng IFN-γ and 10 ng TNF-α treatment induced the activation of GSDMD in the cytoplasm in HT29 and cleaved GSDMD into the P30 fragment, but IFN-γ and TNF-α could not induce the activation of GSDMD in the cell membrane and nucleus in HT29, so it could not form polymers on the cell membrane to induce pyrosis. GSDME was not expressed in HT29. IFN-γ and TNF-α treatment could induce the activation of GSDME in the cytoplasm in 293T and cleave GSDME into the p34 fragment, but it could not induce the activation of GSDME in the cell membrane and nucleus, so it could not induce pyrosis in 293 T. IFN-γ and TNF-α treatment induced activation of caspase-3 in the cytoplasm in 293T, but could not induce activation of caspase-3 in the cell membrane and nucleus in 293T; IFN-γ and TNF-α could not induce the activation of caspase-3 in the cytoplasm, membrane, and nucleus in HT29; IFN-γ and TNF-α treatment induced activation of caspase-8 in the cytoplasm in 293T, but a little level of activation; IFN-γ and TNF-α could not induce activation of caspase-8 in the cell membrane and nucleus in 293T; HT29 did not express caspase-8; thus, IFN-γ and TNF-α treatment did not induce PANoptosis by apoptosis in 293 T or HT29. IFN-γ and TNF-α treatment could not induce the activation of p-MLKL in the cytoplasm, membrane, and nucleus in 293T or HT29, so it could not induce PANoptosis by necrosis. IFN-γ and TNF-α treatment could not induce activation of GSDMD in the cell membrane in HT29 and activation of GSDME in the cell membrane in 293T, so it could not induce pyrosis to induce cell death, could not induce activation of caspase-3 and caspase-8 in 293T or HT29 to induce apoptosis, and could not induce activation of p-MLKL in 293T or HT29 to induce cell necrosis. Therefore, IFN-γ and TNF-α treatment could not induce PANoptosis in 293 T or HT29 (Fig. 5c).

### GSDMD and GSDME oligomerize into pore formation

**Fig. 6:**
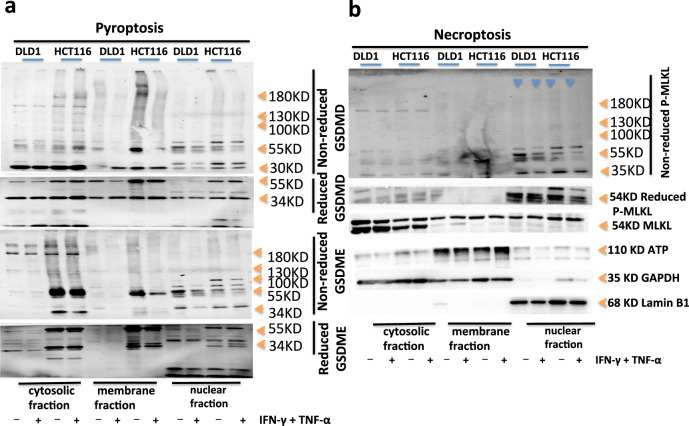
Oligomerization of NT-GSDMD, NT-GSDME, and p-MLKL triggers PANoptosis after co-treatment of IFN-γ + TNF-α. Immunoblot analysis of pro-(P53), activated (P30), oligomerized GSDMD, and pro-(P53), activated (P34), oligomerized GSDME (**a**); tMLKL, phosphorylated MLKL (pMLKL) and oligomerized pMLKL (**b**) induced by PBS or IFN-γ and TNF-α in DLD1 or HCT116; the active NT-GSDMD, NT-GSDME, p-MLKL could form oligomers, 100kD, 130KD, 180KD, and bigger oligomers. The active NT-GSDMD, NT-GSDME mainly in the cytosolic fraction and membrane fraction, little in the nuclear fraction; the active p-MLKL is mainly in the nuclear fraction, little in the cytosolic fraction and membrane fraction. ATP, GAPDH, and Lamin B1 were used as membrane, cytosolic, and nuclear internal control respectively. Data are representative of at least three independent experiments.

To examine GSDMD oligomerization, which correlates with pore formation, we used a protocol that NT-GSDMD distinguishes monomeric and oligomeric GSDMD. Specifically, NT-GSDMD is oligomers under SDS-PAGE electrophoresis without a reducing agent, but oligomers become monomers after adding a reducing agent. The results showed that DLD1 and HCT116 GSDMD were activated by PBS treatment, and cleaved into P30 fragments. There was natural hyperactivation, and the P30 fragments existed in the cytoplasm, membrane, and nucleus. IFN-γ and TNF-α enhanced the activation of GSDMD in the cytoplasm, membrane, and nucleus in DLD1 or HCT116; under the non-reduced SDS-PAGE electrophoresis, the activated P30 fragments of GSDMD were polymers with a molecular weight of more than 400kd in DLD1 or HCT116. The pores of P30 polymers that existed in the cytoplasm and membrane, but not in the nucleus in DLD1, were present in the concentration gel and the separation gel. The pores of P30 polymers existed in the cytoplasm, membrane, and nucleus in HCT116. Noted that GSDMD was activated and cleaved into P30, and the pores of 16 P30 polymers were formed on the cell membrane to induce cell death in DLD1 and HCT116 with the treatment of PBS or IFN-γ and TNF-α. DLD1 did not express GSDME. After the treatment with PBS or IFN-γ and TNF-α on HCT116, GSDME was activated and cleaved into the p34 fragment in HCT116. The p34 fragments existed in the cytoplasm, membrane, and nucleus, and IFN-γ and TNF-α enhanced activation of GSDME in HCT116; under the non-reduced SDS-PAGE electrophoresis, activated p34 fragments of HCT116 GSDME formed polymers with a molecular weight of more than 400 kd. As the pores of 16 P34 polymers that existed in the cytoplasm and membrane, but not in the nucleus in HCT116, were present in the concentration gel and the separation gel, indicating that HCT116 could form polymer pores in the cell membrane and induce cell death (Fig. 6a). p-MLKL in DLD1 or HCT116 with treatment of PBS or IFN-γ and TNF-α could be activated, and the level of activation was not different, but only existed in the nucleus, and formed polymers only in the nucleus, but not on the cell membrane to induce cell necrosis (Fig. 6b). This was also consistent with the previous results that IFN-γ and TNF-α treatment could induce the activation of GSDMD and GSDME in DLD1 or HCT116, inducing the formation of multimeric pores on the cell membrane to promote PANoptosis through pyroptosis and apoptosis, but not necrosis.

### DNA sensors for DNA fragments in cancer cells with dMMR are in the nucleus, not in the cytoplasm

**Fig. 7:**
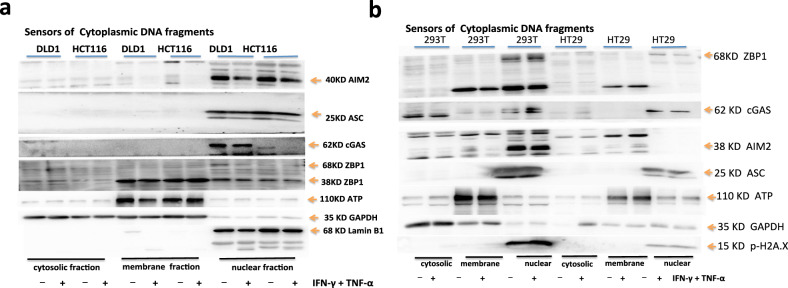
DNA sensors for DNA fragments in Cancer cells with dMMR are in the nucleus, not in the cytoplasm. Immunoblot analysis of cGAS, ZBP1, AIM2, ASC in DLD1, HCT116 (**a**), 293 T or HT29 (**b**). ATP, GAPDH, Lamin B1 were used as membrane, cytosolic, and nuclear internal control respectively. Data are representative of at least three independent experiments.

When MLH1 was absent, cancer cells’ genome was unstable, releasing many DNA fragments and promoting many genes to express. We analyzed the expression and location of DNA sensors in cancer cells. It showed that cGAS, AIM2, ZBP1, or ASC in DLD1 or HCT116 with treatment of PBS or IFN-γ and TNF-α, had no difference in the expression level, but cGAS was only expressed in DLD1 but not in HCT116; after the treatment of IFN-γ and TNF-α in HCT116, lamin B1 was activated and cleaved into a P45 fragment, indicating that IFN-γ and TNF-α activated apoptotic molecules caspase-3 and caspase-7 to degrade the nuclear fiber layer and induce apoptosis; however, after the treatment of PBS in HCT116, it also showed lamin B1 activation and to be cleaved into P45 fragment, indicating that HCT116 had natural nuclear membrane damage, which was consistent with the natural activation of caspase-7 (Fig. 7a); however, in 293T, both the treatment of PBS and IFN-γ plus TNF-α could induce ZBP1 to be expressed, and the level of expression was not different, all of them were only in the nucleus, but ZBP1 was not expressed in HT29, which was consistent with the result that p-MLKL was not naturally activated in HT29; both the treatment of PBS and IFN-γ plus TNF-α could induce cGAS to be expressed in 293 T or HT29 but cGAS only existed in the nucleus in HT29, and cGAS was expressed in the cytoplasm and nucleus in 293T; AIM2 was not expressed in HT29, which indicated that the previous natural hyperactivation of GSDMD could not be activated by AIM2 pathway, but by other pathways; both the treatment of PBS and IFN-γ plus TNF-α could induce AIM2 to be expressed in 293T, AIM2 only existed in the nucleus; in addition, lamin B1 was not activated in 293 T or HT29 and was not cleaved into P45 fragment, which was similar to the previous result that IFN-γ and TNF-α did not induce apoptosis in 293T or HT29 (Fig. 7b).

### Hyperactivation of PANoptosis effective molecules by AIM2-ZBP-RIPK1-RIPK3-ASC-

#### CASP8-CASP1 signal pathway

**Fig. 8:**
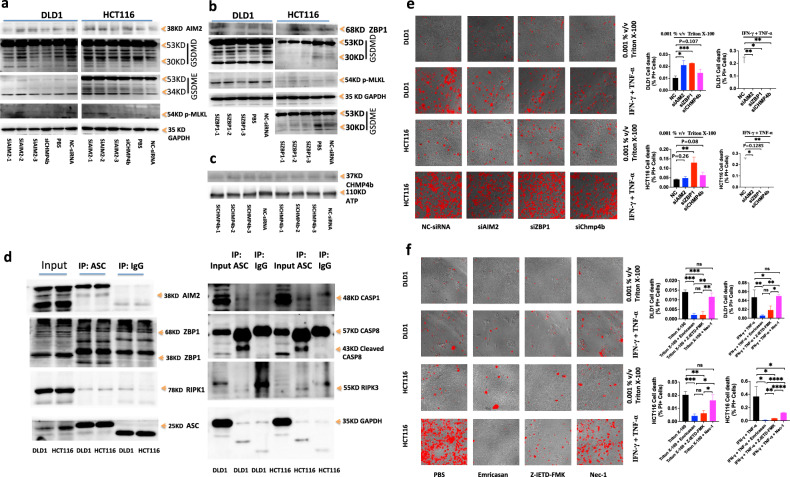
Hyperactivation of PANoptosis effective molecules by AIM2-ZBP1-RIPK1-RIPK3-ASC-CASP8-CASP1 signal pathway. Immunoblot analysis of AIM2, ZBP1, GSDMD, GSDME, p-MLKL in DLDL1 or HCT116 treated with siAIM2s, siZBP1s or siChmp4b (**a**–**c**). Immunoblot analysis of AIM2, ZBP1, RIPK1, ASC, CASP1, CASP8, RIPK3 in DLDL1 or HCT116 immunoprecipitated with IgG control antibodies or anti-ASC antibodies (**d**). GAPDH was used as the internal control. DLD1 and HCT116 were treated by 0.001% v/v Triton X-100 for 7 h or by IFN-γ + TNF-α for 24 h, after treatment respectively with NC-siRNA, siAIM2, siZBP1 or siChmp4b for 48 h. DLD1 and HCT116 were treated with 0.001% v/v Triton X-100 for 12 h after treatment with Emricasan alone, Z-IETD-FMK alone or Nec-1 alone for 24 h (**e**); or DLD1 and HCT116 were co-treated with IFN-γ + TNF-α, IFN-γ + TNF-α + Emricasan, IFN-γ + TNF-α + Z-IETD-FMK or IFN-γ + TNF-α + Nec-1 for 36 h (**f**). All cells were stained by PI, and time-lapse confocal images of PI + DIC. Two-tailed unpaired Student’s *t*-test was used to determine significance (*P < 0.05; **P < 0.01; ***P < 0.001; ****P < 0.0001; NS, not significant). Scale bars, 50 um. Data are representative of at least three independent experiments.

We further investigated the effect of AIM2 or ZBP1 on the hyperactivation of PANoptosis effective molecules by RNA Interference. After treating DLD1 and HCT116 with siAIM2-1 and siAIM2-3 respectively, the activation of GSDMD p30, GSDME p34, and p-MLKL in DLD1 or HCT116 was inhibited, indicating that AIM2 was a receptor for nuclear DNA fragments in DLD1 or HCT116 cells. DLD1 and HCT116 genomes with mismatch repair deficiency were unstable, forming more nuclear DNA fragments. GSDMD p30, GSDME p34, p-MLKL were activated through AIM2-RIPK1-RIPK3-ASC-CASP8-CASP1, GSDMD p30, GSDME p34 were shown in Fig. 5 that they were located in the cytoplasm and membrane, while p-MLKL was located in the nucleus (Fig. 8a). After processing DLD1 and HCT116 with si-ZBP1-3 and si-ZBP1-1 respectively, the result showed that si-ZBP1-3 and si-ZBP1-1 knocking down-the expression of ZBP1 in DLD1 and HCT116 respectively inhibited the activation of GSDMD p30, GSDME p34, and p-MLKL in DLD1 or HCT116, indicating that ZBP1 was a receptor for nuclear DNA fragments in DLD1 and HCT116 cells. The DLD1 and HCT116 genomes with mismatch repair deficiency were unstable, forming more nuclear DNA fragments in cells. GSDMD p30, GSDME p34, and p-MLKL were activated through ZBP1-RIPK1-RIPK3-ASC-CASP8-CASP1. GSDMD p30 and GSDME p34 were located in the cytoplasm and membrane, as shown in Fig. 5, while p-MLKL was located in the nucleus (Fig. 8b). We sought to understand the molecular relationship between AIM2 and ZBP1 in inducing inflammatory cell death and PANoptosis in DLD1 or HCT116. We observed interactions of ASC with AIM2, ZBP1, CASP1, CASP8, RIPK3 and RIPK1 in DLD1 or HCT116 by immunoprecipitation. AIM2-ZBP1-ASC-RIPK1-RIPK3-CASP1-CASP8 could form a PANoptotic complex, activated CASP1 and CASP8 could cleave and activate GSDMD, activated CASP8 could activate CASP3, activated CASP3 could cleave and activate GSDME, and activated p-RIPK3 could directly activate p-MLKL, thereby activating GSDMD p30, GSDME p34, p-MLKL through AIM2-ZBP1-ASC-RIPK1-RIPK3-CASP1-CASP8 PANoptotic complex. The results showed that any knockdown of AIM2 or ZBP1 affected the activation of GSDMD p30, GSDME p34, and p-MLKL, indicating that AIM2 and ZBP1 both acted as DNA receptors and upstream regulators of the PANoptotic complex. The activated GSDMD p30 and GSDME p34 were shown in Fig. 5, located in the cytoplasm and membrane, while p-MLKL was located in the nucleus (Fig. 8d). We further treated AIM2 or ZBP1-knocking down DLD1 and HCT116 with Triton X-100 or IFN-γ and TNF-α to induce cell death. We found that compared to the control siRNA, knocking down the expression of AIM2 and ZBP1 genes could increase cell death induced by Triton X-100 in DLD1 and HCT116, but reduce cell death induced by IFN-γ and TNF-α (Fig. 8e). It confirmed that reducing the membrane repair activity by targeting the molecular in the dsDNA-AIM2-ZBP1-ASC-RIPK1-RIPK3-CASP1-CASP8-GSDMD-GSDME pathway could increase the sensitivity of tumor cell membranes to Triton X-100, but reduce the sensitivity to IFN-γ and TNF-α. At the same time, we found that knockdown of Chmp4b gene expression increased Triton X-100-induced PANoptotic cell death in DLD1 and HCT116, but inhibited IFN-γ and TNF-α-induced PANoptotic cell death (Fig. 8c, e). Cell death induced by Triton X-100 or IFN-γ and TNF-α in DLD1 and HCT116 needed the activation of caspase, but not the p-RIPK1 activity except for IFN-γ and TNF-α-induced cell death in HCT116 (Fig. 8f).

### The aggregation of N-GSDMD or N-GSDME is in the nucleus

**Fig. 9:**
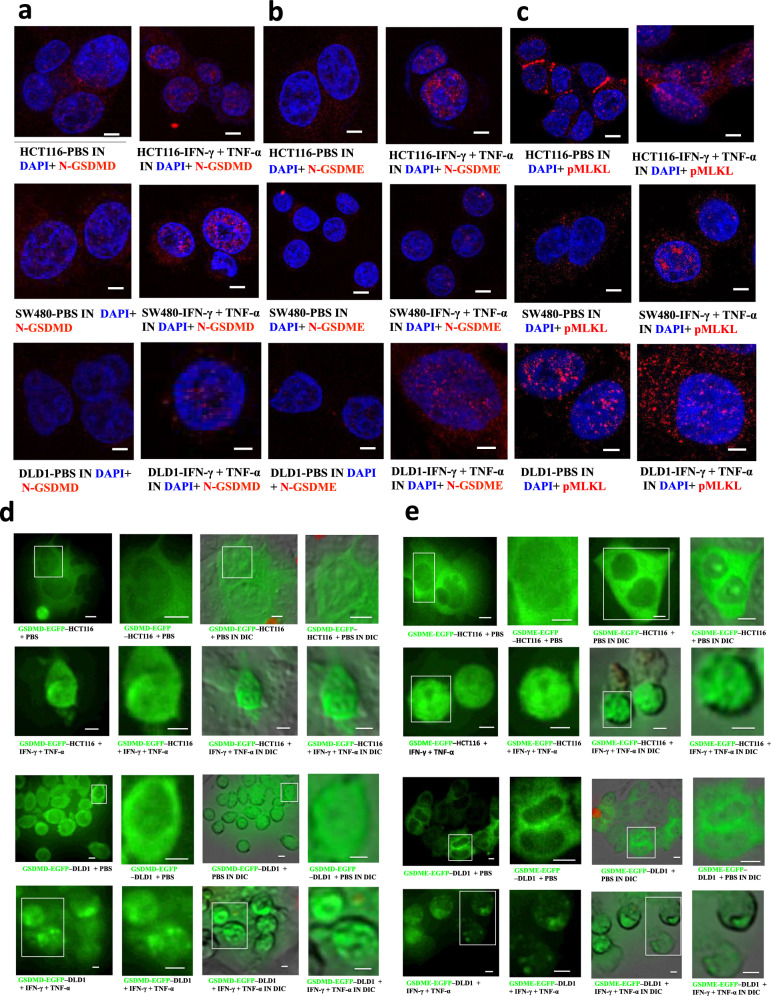
Location of PANoptotic molecules induced by co-treatment of IFN-γ + TNF-α using immunofluorescence and time-lapse confocal microscopy. Fixed-cell microscopy images of NT-GSDMD(red), NT-GSDME(red), p-MLKL (red) in HCT116, SW480, DLD1 treated with PBS or IFN-γ + TNF-α (**a**, **b**, **c**) and time-lapse confocal images of either GSDMD-EGFP–expressing or GSDME-EGFP–expressing DLD1 or HCT116 cells after a 24-h PBS or TNF-α + IFN-γ treatment (**d**, **e**). Nucleus stained by DAPI (blue), DIC, differential interference contrast. Results are representative of at least three independent experiments. Scale bars, 5 mm.

Next, we sought to find out how pyroptotic molecules induced cell death after co-treatment of IFN-γ and TNF-α by time-lapse confocal images. The results showed that GSDMD-EGFP or GSDME-EGFP mainly existed in the cytoplasm and cell membrane in DLD1 or HCT116, and after the treatment of IFN-γ and TNF-α, most of them formed bright spots in the nucleus and formed aggregates, which was consistent with the previous result that the aggregation of N-GSDMD or N-GSDME was in the nucleus after the treatment of IFN-γ and TNF-α (Fig. 9a–c). In addition, GSDMD-EGFP-expressing-DLD1 or GSDMD-EGFP, or GSDME-EGFP-expressing-HCT116 mostly had cell membrane and nucleus rupture. One of the reasons was that GSDMD-EGFP and GSDME-EGFP were induced to aggregate in the nucleus after cell death (Fig. 9d, e).

### Triton X-100 treatment on SW620, DLD1, HCT116 or HT29 induces cell outer membrane damage

**Fig. 10:**
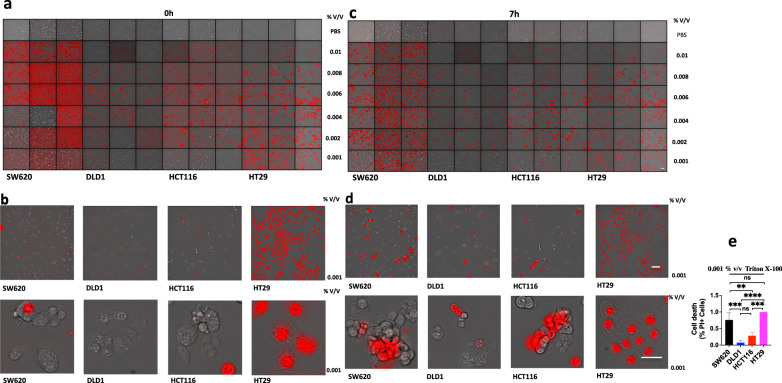
Triton X-100 induces lysis of cell membranes. SW620, DLD1, HCT116, or HT29 were treated by Triton X-100 at 0.01, 0.008, 0.006, 0.004, 0.002, 0.001% v/v for 0 h, 7 h (**a**–**d**). All cells were stained by PI, and time-lapse confocal images of PI + DIC. One-way ANOVA was used to determine significance (*P < 0.05; **P < 0.01; ***P < 0.001; ****P < 0.0001; NS, not significant) (**e**). Scale bars, 50 µm. Data are representative of at least three independent experiments.

A high concentration of Triton X-100 can lyse cells, and a low concentration of Triton X-100 can increase cell membrane permeability. Triton X-100 is used to simulate the effect molecules of T cells killing tumor cells, such as perforin and granzyme. When treatment with Triton X-100 at concentrations of 0.01, 0.008, 0.006, 0.004, or 0.002% v/V in SW620, DLD1, HCT116, or HT29, it could immediately induce a large number of cell death. However, when treatment with Triton X-100 at the concentration of 0.001% v / V, it could immediately induce a large number of cell death in HT29, but it could induce little cell death in SW620, DLD1, or HCT116 (Fig. 10a, b). When treated with Triton X-100 at the concentration of 0.001% v / V for 7 h, it could induce a large number of cell death in SW620 or HT29, but it could induce little cell death in DLD1 or HCT116, and SW620 and HT29 are mismatch repair proficient cell lines, but DLD1 and HCT116 are mismatch repair deficient cell lines (Fig. 10c–e). The reason was that DLD1 and HCT116 (dMMR) led to the formation of a large number of DNA fragments, activating AIM2-ZBP1-CASP1-CASP8-GSDMD-GSDME pathway to form pores on the cell membrane and causing cell membrane damage, finally inducing the membrane repair system. Therefore, DLD1 and HCT116 (dMMR) were not sensitive to the low concentration of membrane damage agent Triton X-100, while SW620 and HT29 (pMMR) did not induce membrane repair system so they were sensitive to cell membrane damage at the low concentration of Triton X-100 [12].

### *Mlh1* knockout CT26 and CMT93 undergo natural hyperactivation of GSDMD, GSDME and p-MLKL

**Fig. 11:**
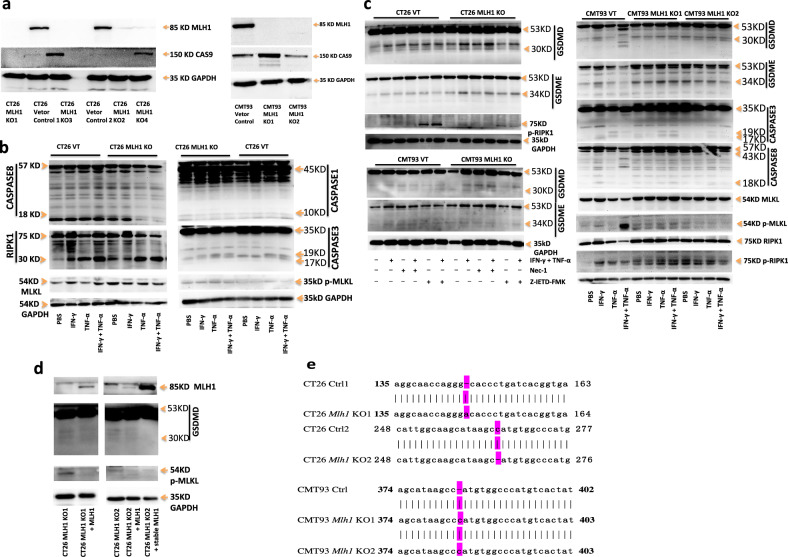
*Mlh1* knockout CT26 and CMT93 undergo natural hyperactivation of GSDMD, GSDME, and p-MLKL. Immunoblot analysis of MLH1, CAS9 in CT26 and CMT93 Vector Control, CT26 and CMT93 MLH1 KO (**a**); pro-(P53) and activated (P30) GSDMD, pro-(P53) and activated (P34) GSDME, pro-(P45) and activated (P10) CASP1, pro-(P35) and cleaved (P19 and P17) CASP3, pro-(P55) and cleaved (P18) CASP8, total MLKL (tMLKL), p-MLKL, p-RIPK1, and pro- (P75) and cleaved (P30) RIPK1 in CT26 and CMT93 Vector Control, CT26 and CMT93 MLH1 KO, after treatment with IFN-γ alone, TNF-α alone or IFN-γ + TNF-α for 96 h or treatment with Nec-1 alone, Z-IETD-FMK alone, IFN-γ + TNF-α, IFN-γ + TNF-α + Nec-1 or IFN-γ + TNF-α + Z-IETD-FMK for 24 h (**b**, **c**); MLH1, pro-(P53) and activated (P30) GSDMD, p-MLKL in CT26 MLH1 KO1 + *Mlh1*, CT26 MLH1 KO2 + *Mlh1* and CT26 MLH1 KO2 + stable *Mlh1* (**d**). **e** genome sequencing was used to identify alterations of the *Mlh1* sequence in the indicated cell clones. The effects of these changes were frameshifts. GAPDH was used as the internal control. Data are representative of at least three independent experiments.

We further investigated the hyperactivation of PANoptotic molecules in dMMR CT26 and CMT93 compared with pMMR CT26 and CMT93 (Fig. 11a). Comparing GSDMD and GSDME in pMMR CT26 and CMT93 with the treatment of PBS, GSDMD and GSDME in CT26 and CMT93 *Mlh1* KO with the treatment of PBS were natural hyperactivation and cleavage of P30 and P34 fragments, and treatment with IFN-γ and TNF-α enhanced the activation of GSDMD and GSDME and induced pyroptotic cell death (Fig. 11c). We also found that there was natural hyperactivation of caspase-1, caspase-3, caspase-8 and cleavage of RIPK in CT26 *Mlh1* KO with treatment of PBS and natural hyperactivation of caspase-3, caspase-8 and phosphorylation of RIPK1 in CMT93 *Mlh1* KO with treatment of PBS, and treatment with IFN-γ and TNF-α enhanced the activation of these PANoptotic molecules, except for phosphorylation of RIPK1 in CMT93 *Mlh1* KO. Z-IETD-FMK could inhibit natural hyperactivation and IFN-γ and TNF-α-enhancing-activation of GSDMD and GSDME in CT26 and CMT93 *Mlh1* KO, but Nec-1 could not. There was no or little activation of these PANoptotic molecules in CT26 and CMT93 VT with treatment of PBS and treatment with IFN-γ and TNF-α induced weaker activation of these PANoptotic molecules than that in CT26 and CMT93 *Mlh1* KO (Fig. 11b, c). p-MLKL in CT26 and CMT93 *Mlh1* KO also showed significant natural hyperactivation with treatment of PBS, compared with that in CT26 and CMT93 VT, and the activation level was no significant difference among the treatment of PBS, TNF-α, IFN-γ or TNF-α and IFN-γ in CT26 and CMT93 *Mlh1* KO; at the same time, p-MLKL in CT26 and CMT93 VT with treatment of PBS had also weak natural hyperactivation, the activation level was no significant difference among the treatment of PBS, TNF-α, IFN-γ or TNF-α and IFN-γ in CT26 VT, but TNF-α and IFN-γ could enhance activation of p-MLKL in CMT93 VT (Fig. 11b, c). It indicated that CT26 and CMT93 *Mlh1* KO led to the formation of more DNA fragments, thus activating the cytoplasmic receptors, such as cGAS, AIM2, and ZBP1. However, our previous results showed that these receptors existed in the nucleus, in which the nuclear dsDNA-AIM2-ZBP1- RIPK3-p-MLKL pathway activated p-MLKL and locking it in the nucleus, losing activation from the external signal stimulation, and leading to escape from cell necrosis. At the same time, there was also natural hyperactivation of dsDNA-AIM2-ZBP1-CASP1-CASP8-GSDMD-GSDME. At the same time, we found that CT26 and CMT93 VT also exhibited natural hyperactivation of GSDMD, GSDME, and p-MLKL, because they were constructed by cas9-expressing lentiviral vector, they could induce natural hyperactivation of GSDMD, GSDME, and p-MLKL through viral infection. This was also the same conclusion of other studies that influenza virus infection can naturally activate ZBP1 [13]. However, our experimental results showed that the natural hyperactivation of GSDMD, GSDME, and p-MLKL in CT26 and CMT93 *Mlh1* KO was stronger than that in CT26 and CMT93 VT (Fig. 11b, c). The relevant conclusions can be further verified by the experiment of nonviral vector *Mlh1* KO cell. We restored CT26 *Mlh1* KO using transient or stable overexpression of the *Mlh1* gene. Compared with CT26 *Mlh1* KO, the natural hyperactivation of GSDMD and p-MLKL in *Mlh1* rescued CT26 *Mlh1* KO disappeared, confirming that the natural hyperactivation of GSDMD and p-MLKL was closely related to *Mlh1* gene expression (Fig. 11d).

### Triton X-100 treatment on *Mlh1* KO CT26 and CMT93 induces cell outer membrane damage

**Fig. 12:**
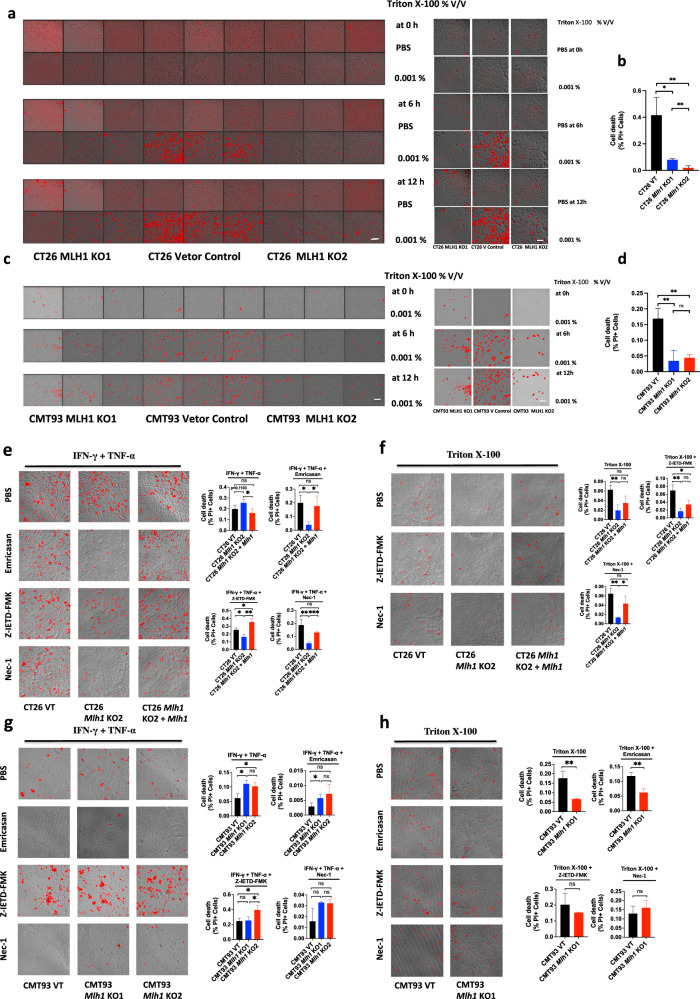
Triton X-100 or IFN-γ and TNF-α induces PANoptosis in CT26 and CMT93 MLH1 KO or CT26 and CMT93 Vector Control. CT26 and CMT93 MLH1 KO, CT26, and CMT93 Vector Control were treated by Triton X-100 at 0.001% v/v for 0 h, 6 h, or 12 h (**a**–**d**); CT26 and CMT93 Vector Control, CT26 and CMT93 MLH1 KO or CT26 MLH1 KO + *Mlh1* were co-treated with IFN-γ + TNF-α, IFN-γ + TNF-α + Emricasan, IFN-γ + TNF-α + Z-IETD-FMK or IFN-γ + TNF-α + Nec-1 for 36 h (**e**, **g**); or treated with 0.001% v/v Triton X-100 for 12 h after treatment with Emricasan alone, Z-IETD-FMK alone or Nec-1 alone for 24 h (**f**, **h**). All cells were stained by PI and time-lapse confocal images of PI + DIC. Two-tailed unpaired Student’s *t*-test was used to determine significance (*P < 0.05; **P < 0.01; ***P < 0.001; NS, not significant). Scale bars, 50 um. Data are representative of at least three independent experiments.

As shown above that *Mlh1* KO CT26 and CMT93 can repeat the natural hyperactivation of GSDMD, GSDME, or p-MLKL in DLD1, HCT116 (dMMR), SW620, or HT29 (pMMR), so they can also repeat the sensitivity to Triton X-100. Triton X-100 at the concentration of 0.001% v / V was used to treat CT26 and CMT93 *Mlh1* KO1, CT26 and CMT93 Vector Control or CT26 and CMT93 *Mlh1* KO2 for 6 h or 12 h, it could induce a large number of cell death in CT26 and CMT93 Vector Control, while it only induced little cell death in CT26 and CMT93 *Mlh1* KO1 or CT26 and CMT93 *Mlh1* KO2 (Fig. 12a–d). This showed that CT26 and CMT93 cells with *Mlh1* gene knockout caused a large number of DNA fragments, which caused cell membrane damage through the AIM2-ZBP1-CASP1-CASP8-GSDMD-GSDME pathway, inducing the membrane repair mechanism, so that they were insensitive to the membrane damage agent Triton X-100. However, p-MLKL was mainly activated in the nucleus in CT26 and CMT93 *Mlh1* KO, so it could not damage the cell membrane and induce cell necrosis. In conclusion, the membrane damage induced by *Mlh1* knockout activated the membrane repair system through the pyroptotic molecules GSDMD and GSDME, rather than the necrotic molecule p-MLKL.

Compared with CT26 VT, CT26 *Mlh1* KO reproduced cell death induced by IFN-γ plus TNF-α or Triton X-100 in DLD1 and HCT116, CT26 *Mlh1* KO increased IFN-γ and TNF-α-induced cell death, and reduced cell death induced by Triton X-100. CT26 *Mlh1* KO was sensitive to pan-caspase inhibitor Emricasan, caspase-8 inhibitor Z-IETD-FMK, and RIPK1 inhibitor Nec-1, all of which could inhibit cell death induced by IFN-γ plus TNF-α or Triton X-100. *Mlh1* rescued CT26 *Mlh1* KO restored the characteristics of CT26 VT, restored the sensitivity to IFN-γ plus TNF-α or Triton X-100, and insensitivity to Emricasan, Z-IETD-FMK and RIPK1 inhibitor Nec-1 (Fig. 12e, f). Compared with CMT93 VT, CMT93 *Mlh1* KO reproduced cell death induced by IFN-γ plus TNF-α or Triton X-100 in DLD1 and HCT116, increasing IFN-γ and TNF-α-induced cell death and reducing cell death induced by Triton X-100, but exhibited different sensitivities to Emricasan, Z-IETD-FMK, and Nec-1. Both Emricasan and Nec-1 could inhibit cell death induced by IFN-γ and TNF-α in CMT93 VT and CMT93 *Mlh1* KO, and Z-IETD-FMK increased IFN-γ + TNF-α-induced cell death in CMT93 VT and CMT93 *Mlh1* KO. There was no difference in cell death affected by Emricasan, Z-IETD-FMK, or Nec-1 in CMT93 VT and CMT93 *Mlh1* KO; Z-IETD-FMK and Nec-1 significantly increased cell death induced by Triton X-100 in CMT93 *Mlh1* KO, while Z-IETD-FMK slightly increased cell death induced by Triton X-100 in CMT93 VT, but Nec-1 inhibited Triton X-100-induced cell death in CMT93 VT. Emricasan had little effect on Triton X-100-induced cell death in CMT93 VT and CMT93 *Mlh1* KO (Fig. 12g, h).

### *Mlh1* knockout cells induce a strong immune reaction to PANopotosis

**Fig. 13:**
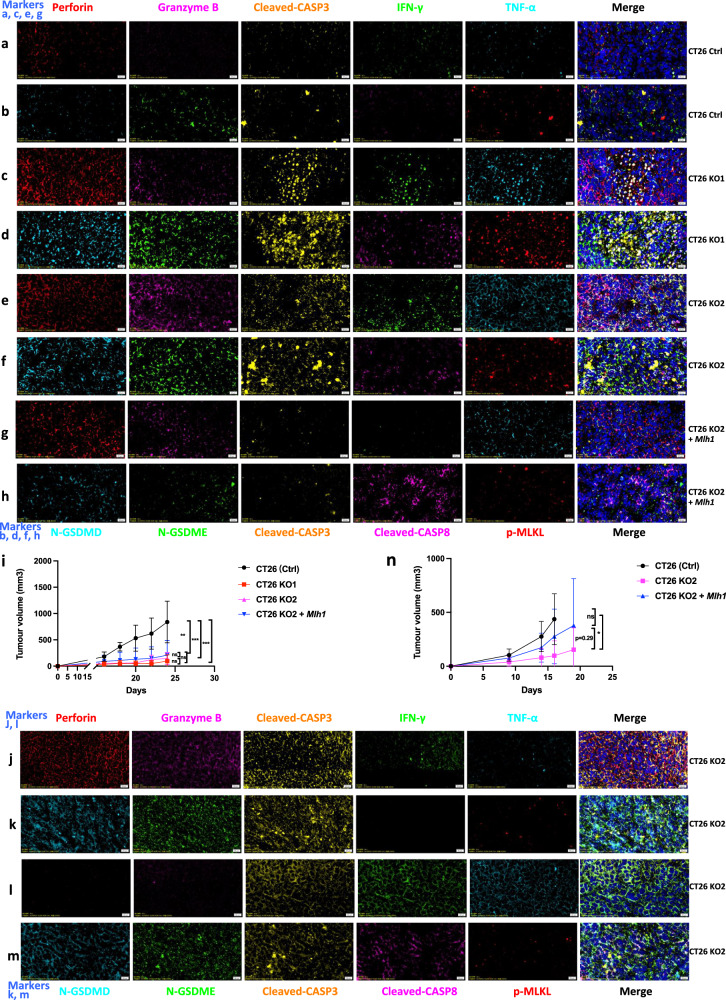

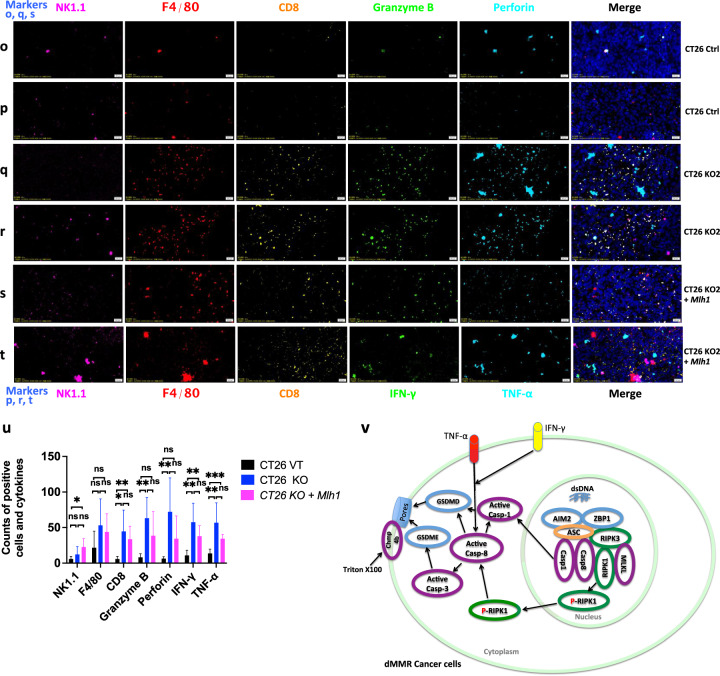
Immune microenvironment in tumors generated with CT26 (Ctrl), CT26 KO1, CT26KO2, or CT26 KO2 + *Mlh1.* **a**–**m**, **o**–**t** 2 × 10^5^ CT26 (Ctrl), CT26 KO1, CT26KO2, or CT26 KO2 + *Mlh1* were injected 180 days after genome editing into syngeneic BALB/c mice at the indicated time points (*n* = 6) (**i**); Multiplex immunohistochemistry of Perforin, Granzyme B, Cleaved-CASP3, IFN-γ, TNF-α, and DAPI (**a**, **c**, **e**, **g**, **j**, **l**); N-GSDMD, N-GSDME, Cleaved-CASP3, Cleaved-CASP8, p-MLKL, and DAPI (**b**, **d**, **f**, **h**, **k**, **m**); NK.1.1, F4/80, CD8, Perforin, Granzyme B, and DAPI (**o**, **q**, **s**); NK.1.1, F4/80, CD8, IFN-γ, TNF-α, and DAPI (**p**, **r**, **t**) in the CT26 (Ctrl), CT26 KO1, CT26KO2 or CT26 KO2 + *Mlh1* tumor samples from **i**. The images are representative sections from one mouse. The staining was performed on six independent mice. **n** 10^6^ CT26 clones were injected into syngeneic BALB/c mice (*n* = 7). Counts of NK1.1, F4/80, CD8+, Perforin, Granzyme B, IFN-γ, and TNF-α per high-power field from representative fields from each experimental arm (CT26 (Ctrl) group (*n* = 6), CT26KO2 group (*n* = 5) and CT26 KO2 + *Mlh1* group (n = 4)). Experiments were repeated at least two times. Data are presented as mean ± SEM. Statistical analysis was performed using one-way ANOVA (**i**, **n**) and two-tailed unpaired Student’s *t*-test (**u**) (*P < 0.05; **P < 0.01; ***P < 0.001; NS, not significant). Ctrl, control. Scale bars, 20 μm. **v**, Schematic of dsDNA fragments driven hyperactivation of pyroptosis and IFN-γ and TNF-α-induced cell death in dMMR cancer cells.

After subcutaneous injection into immunocompetent syngeneic mice (BALB/c) with 2 × 10^5^ mouse tumor cell lines CT26 (Ctrl), CT26 KO1, CT26 KO2, or CT26 KO2 + *Mlh1*, tumor size was measured twice a week. After one month, the tumor growth curve in the mice was analyzed. We found that the tumor volume formed by CT26 (Ctrl) was the largest, with an average of over 1000 mm^2^. The tumor volume formed by CT26 KO1, CT26 KO2, or CT26 KO2 + *Mlh1* was about 200 mm^2^, compared to the CT26 (Ctrl) group, the difference was significant (Fig. 13i). We further analyzed the immune microenvironment of tumor formation in various cell lines. In the tumor microenvironment formed by CT26 (Ctrl), immune effector molecules perforin, granzyme B, IFN-γ, and TNF-α were rarely expressed, and the PANoptotic molecules N-GSDMD, N-GSDME, cleaved-CASP3, cleaved-CASP8, and p-MLKL were also rarely expressed; comparison of CT26 (Ctrl) tumor microenvironment, CT26 KO1, CT26 KO2 tumor microenvironment highly expressed immune effector molecules perforin, granzyme B, IFN-γ, TNF-α and induced more expression of PANoptotic molecules N-GSDMD, N-GSDME, cleaved-CASP3, cleaved-CASP8, and p-MLKL, leading to significant differences in tumor volume. Meanwhile, tumors formed by CT26 (Ctrl) and CT26 KO also reproduced the scene that the expression of N-GSDMD, N-GSDME, and p-MLKL was induced in vitro in CT26 (Ctrl) and CT26 KO cells by IFN-γ + TNF-α co-treatment. p-MLKL in CT26 KO tumor tissue was mainly locked in the nucleus, while N-GSDMD and N-GSDME mainly existed in the cytoplasm and cell membrane (Fig. 13a–f). Tumors formed by CT26 (Ctrl) lacked immune effector molecules perforin, granzyme B, IFN-γ, TNF-α, and induced few PANopotosis effector molecules N-GSDMD, N-GSDME, and p-MLKL. High mutation of CT26 KO cells induced activation of large immune cells in mice, aggregating around CT26 KO tumors and releasing large of inflammatory factors such as perforin, granzyme B, IFN-γ and TNF-α, which induced the activation of PANopotosis effector molecules N-GSDMD, N-GSDME, cleaved-CASP3, cleaved-CASP8, and p-MLKL that inhibited CT26 KO tumor growth, while CT26 (Ctrl) had low immunogenicity and induced few activated immune cells and inflammatory factors, which could not inhibit tumor growth.

We found that perforin, granzyme B, IFN-γ, and TNF-α were unevenly distributed within the tumor, showing that these were three pathways to induce activation of N-GSDMD, N-GSDME, and cleaved-CASP3 by perforin, granzyme B, IFN-γ, and TNF-α. Three scenarios to induce activation of N-GSDMD, N-GSDME, and cleaved-CASP3: (1) perforin, granzyme B, IFN-γ, and TNF-α co-induce the activation of N-GSDMD, N-GSDME, and cleaved-CASP3; (2) perforin and granzyme B co-induce the activation of N-GSDMD, N-GSDME, and cleaved-CASP3; (3) IFN-γ and TNF-α co-induce the activation of N-GSDMD, N-GSDME, and cleaved-CASP3, which was also consistent with our previous results that perforin and granzyme B attacked and disrupted the tumor cell membrane outside the tumor, IFN-γ and TNF-α activated the PANoptotic pathway within tumor cells and cooperatively inhibited tumor growth (Fig. 13e, f, j–m).

At the same time, we compared the tumor volume formed by CT26 KO2 and CT26 KO2 + *Mlh1* in mice after 1 month, and the difference was not significant (Fig. 13i). We further analyzed the immune microenvironment of the tumor. Tumors formed by CT26 KO2 + *Mlh1* induced partial expression of perforin, granzyme B, and TNF-α and partial activation of N-GSDMD and N-GSDME, which also inhibited tumor growth in CT26 KO2 + *Mlh1*, with little difference in tumor volume compared to CT26 KO (Fig. 13e–h). However, this was inconsistent with other studies reporting significant differences in tumor volume between 4T1 *Mlh1* KO and 4T1 *Mlh1* KO + *Mlh1*. We increased the number of injected tumor cells and subcutaneously injected 1 × 10^6^ CT26 KO2 and CT26 KO2 + *Mlh1* into immunocompetent syngeneic mice. After one month, we found significant differences in tumor volume between the two groups (Fig. 13n). At the same time, we observed partial expression of perforin, granzyme B, and TNF-α and partial activation of N-GSDMD and N-GSDME in tumors formed by CT26 KO2 + *Mlh1*, but a lack of IFN-γ. IFN-γ is an important inflammatory factor that inhibits tumor growth and activates T cells, macrophages, and NK cells. The lack of IFN-γ can lead to a transition from an anti-tumor microenvironment to a pro-tumor microenvironment, and also lead to the depletion of immune-killing cells, thereby promoting tumor growth (Fig. 13e–h).

In addition, through multiplex immunohistochemistry localization, we found that IFN-γ and TNF-α were mainly released by macrophages and CD8+T cells, perforin and granzyme B were released by CD8 + T cells rather than NK cells. At the same time, we found that the number of perforin, granzyme B, IFN-γ, and TNF-α released from between CT26 (Ctrl) and CT26 KO tumor tissue was significant. The number of inflammatory factors released by CT26 KO2 was more than that of CT26 KO2 + *Mlh1* tumor tissue, but no significant difference statistically. At the same time, we observed the co-localization of macrophages and CD8 + T cells in CT26 KO tumor tissue, which resulted in macrophages continuously activating CD8 + T cells as antigen-presenting cells. This phenomenon did not exist in CT26 (Ctrl) tumor tissue (Fig. 13o–u).

## Discussion

The co-treatment of IFN-γ and TNF-α can induce PANoptosis including pyroptosis, apoptosis and necrosis, which can lead to a higher cell death rate. At the same time, our result showed that the cell death rate induced by co-treatment of IFN-γ and TNF-α in DLD1, HCT116, and RKO cells with mismatch repair deficiency was higher than that in SW480, SW620, and HT29 cells with mismatch repair proficiency, and the difference was significant, which inspired us to study the mechanism. In DLD1 or HCT116, we could clearly see IFN-γ and TNF-α-induced significant activation of PANoptotic molecules, including pyroptotic molecules GSDMD, GSDME, apoptotic molecules caspase-3, caspase-7, caspase-8 and necrotic molecule p-MLKL, while GSDMD, GSDME were activated in the cytoplasm, caspase-3, caspase-7, caspase-8 were mainly activated in the cytoplasm, while p-MLKL was mainly activated in the nucleus, and there was no difference in the activation level of p-MLKL induced by between treatment of PBS and IFN-γ and TNF-α, indicating that p-MLKL is locked in the nucleus, and cell death can not be induced by necrosis, but can only be induced by pyroptosis and apoptosis. Compared with HT29 (mismatch repair proficiency), we found that HT29 had multiple defects in cell death pathways, GSDME was not expressed, caspase-8 was not expressed, and AIM2 was not expressed, which also explains that HT29 has no effect on treatment of IFN-γ and TNF-α, and is insensitive to cell death induced by treatment of IFN-γ and TNF-α, treatment of IFN-γ and TNF-α could induce activation of HT29 GSDMD and cleaved it into p30 fragment, but active p30 fragment only existed in the cytoplasm but not in the membrane, so that it cannot induce pyroptotic cell death and it can only induce weak activation of caspase-3 and caspase-8, so it can not induce apoptotic cell death, nor can it activate p-MLKL to induce necrotic cell death, so it can not induce cell death by PANoptosis.

In addition, DLD1 or HCT116 cells with mismatch repair deficiency released exonuclease 1, leading to increased single-strand DNA formation, depletion of RPA, DNA breakage, and abnormal DNA repair intermediates, eventually leading to chromosome abnormalities and nuclear DNA release [11], which induced the hyperactivation of AIM2-ZBP1-ASC-RIPK1-RIPK3-CASP1-CASP8-GSDMD-GSDME [14], leading to cell membrane damage, and inducing membrane repair system [15], therefore, it is insensitive to the low concentration of cell membrane damaging agent Triton X-100, which leads to the production of resistance against T cell killing and immune evasion [12], but we can use IFN-γ and TNF-α to treat DLD1 and HCT116 to induce PANoptosis to overcome the immune escape of tumor cells; SW620 and HT29 (pMMR) do not have this mechanism, so they are sensitive to the low concentration of cell membrane damage agent Triton X-100, and they can be cleared by enhancing tumor immunity.

## Methods

### Cell culture

The human colorectal cancer cell lines DLD1, HCT116, RKO, SW480, SW0620, and mouse colorectal cancer cell lines CT26 (ATCC) were cultured in RPMI media (Corning, 10-040-CV) supplemented with 10% FBS and 1% penicillin and streptomycin. The human colorectal cancer cell lines HT29, embryonic kidney 293T cells, and mouse MC38 were cultured in DMEM media (Corning, 10-040-CV) supplemented with 10% FBS and 1% penicillin and streptomycin.

### Plasmids

To knockout *Mlh1*, we used the gene-editing crispr-cas9 system (lentiCRISPR-v2) (Addgene #52961). sgRNAs were designed using the CRISPR tool (http://crispr.mit.edu) to minimize potential off-target effects. Two sgRNAs, sgRNA1: TCACCGTGATCAGGGTGCCC and sgRNA2: ATTGGCAAGCATAAGCCATG to target mouse *Mlh1*, were cloned into lentiCRISPR-v2 plasmid. Human GSDMD-EGFP, GSDME-EGFP, and mouse *Mlh1* lentiviral vectors were purchased from (Igebio, China).

### Lentivirus production

Lentiviral particles were generated in HEK293T with co-transfection of the above lentiCRISPR-v2, human GSDMD-EGFP, GSDME-EGFP or mouse *Mlh1* lentiviral vector and packaging plasmids pCMV-VSV-G (Addgene #8454) and psPAX2 (Addgene #12260) by Lipofectamine 3000 (Life technologies). Supernatant from HEK293T with transfection of Lentiviral packaging plasmids was collected, passed through a 0.22 μm filter to remove cell debris, and frozen at −80 °C.

### Establishment of stable expression cell lines

For generating stable expression of human GSDMD-EGFP or GEDME-EGFP in DLD1 or HCT116, lentivirus particles were added to cell culture for 48 h, infected cells were selected by 10 μg/mL Puromycin (MP Biomedicals) for two days.

### Gene editing

Mouse colorectal cancer cell lines CT26 and CMT93 were infected with the above *Mlh1* sgRNA-

CRISPR-Cas9-expressing lentivirus at approximately 60% confluence in the presence of 5 μg/mL polybrene (Solarbio, China). 10 μg/mL Puromycin (MP Biomedicals) was used to select infected cells. Two days after selection for Puromycin, infected CT26 cells were single-cell cloned in 96-well plates by flow cytometry using the Beckman Coulter MoFlo XDP cell sorter. *Mlh1*-knockout clone was verified by western blot and DNA sequencing.

### Cell stimulation

For induction of cell death, 10 ng/mL of TNF-α (Peprotech, AF-300-01A), 100 ng/mL of IFN-γ (Peprotech, 300-02), 0.01, 0.008, 0.006, 0.004, 0.002% or 0.001% v/V of Triton X-100 was used for the indicated time. For the inhibition of cell death, cells were co-treated with 20 μM of IDN-6556 (Sellck), 20 μM of Z-IETD-FMK (Sellck), 50 μM of Nec-1 (Sellck), 20 μM of VX-765 (Sellck), 20 μM of Necrosulfonamide (Sellck).

### Cytotoxicity assay

Relevant cells were treated as indicated. Cell viability was determined by the Cell Counting Kit-8 (Dojindo Laboratories). Cell death was measured by the LDH assay using CytoTox 96 Non-Radioactive Cytotoxicity Assay kit (Promega).

### Real-time imaging for cell death

The kinetics of cell death were determined using the ImageXpress Pico (Molecular Devices) live-cell automated system. Cells (2 × 10^3^ cells/well) were seeded in 96-well tissue culture plates. Cells were treated with the indicated cytokines and inhibitor inhibitor, then stained with propidium iodide (Sigma). The plate was scanned, and fluorescent and phase-contrast images (5 image fields/well) were acquired in real-time every 1 h from 0 to 96 h post-treatment. The images were analyzed using the software package supplied with the ImageXpress Pico.

### Subcellular fractionation

According to the manufacturer’s instructions (Abcam), cells were seeded in 100 mm tissue culture plates and grown to semi-confluent density. Cells were detached by treatment with 2 mL of 0.25% Trypsin-EDTA and added into the saved medium. Cells were collected by centrifugation for 5 min at 300 x *g* at RT. Cell pellets were resuspended in 1x Buffer A to 6.6 × 10^6^ cells/mL. An equal volume of Buffer B was added to the cell suspensions and mixed by pipetting. Samples were incubated for 7 min on a rotator at RT and centrifuged at 5000 x *g* for 1 min at 4 °C. All supernatants were carefully removed and re-centrifuged at 10,000 x *g* for 1 min for the cytosolic fractions (C). The sequential cytoplasm-depleted cell pellets were resuspended and combined in 1x Buffer A. Exactly the same volume of Buffer C was added to the suspensions and mixed by pipetting. Samples were incubated for 10 min on a rotator at RT. Samples were centrifuged at 5000 x *g* for 1 min at 4 °C. All supernatants were carefully removed and re-centrifuge the supernatant fractions at 10,000 x *g* for 1 min for the mitochondrial fractions (M). The sequential cell pellets were resuspended and combined in 1x Buffer A to the original volume of suspensions for the nuclear fractions (N). Samples were prepared by adding an appropriate amount of 5X SDS loading buffer and heated to 95 °C for 10 min for immunoblotting.

### Immunoprecipitation

Cells were lysed in a RIPA buffer containing (50 mM Tris(pH7.4), 150 mM NaCl, 1% NP-40, 0.25% sodium deoxycholate, protease inhibitors (Fudebio), phosphatase inhibitors (Fudebio)). After centrifugation at 20,000 x *g* for 10 min, the supernatant was incubated with either IgG control antibody (sc-3877, Santacruz) or anti-ASC antibody (67494-1-Ig, Proteintech) with protein A/G PLUS-Agarose (Santacruz) overnight at 4 °C. After washing with the above lysis buffer, the immunoprecipitated proteins were collected by centrifugation at 20,000 x *g* for 10 min, resuspended and boiled in 1× SDS loading buffer at 95 °C for 10 min.

### Immunoblot analysis

For immunoblot analysis, cells were lysed in RIPA buffer (containing protease inhibitors (Fudebio), and phosphatase inhibitors (Fudebio)). Cells were centrifuged at 20,000 x *g* for 15 min at 4 °C, the soluble fraction was collected. Protein concentrations of cell lysates were determined using a BCA Protein Assay Kit (BeyotimeBio). Samples were prepared by adding an appropriate amount of 5x SDS loading buffer and heated to 95 °C for 10 min for immunoblotting. Proteins were separated by electrophoresis through 12% polyacrylamide gels. Following electrophoretic transfer of proteins onto PVDF membranes (Roche), nonspecific binding was blocked by incubation with 5% BSA, then membranes were incubated with primary antibodies against: caspase-3 (14220, Cell Signaling Technology (CST)), cleaved caspase-3 (9664, CST), caspase-8 (9746, 4790, CST), cleaved caspase-8 (8592, CST), caspase-1 (ab179515, Abcam), GAPDH (60004-1-Ig, Proteintech), pMLKL (37333, CST, ab187091, Abcam), tMLKL (14993, 37705 CST), pRIPK1 (65746, 53286 CST), tRIPK1 (3493, CST), RIPK3 (13526, CST), pRIPK3 (93654, CST), Cas9 (65832, CST), GSDMD (ab209845, Abcam, 39754, CST), GSDME (ab215191, Abcam), MLH1 (ab92312, Abcam), cGAS (31659, 15102, CST), ZBP1 (13285-1-AP, Proteintech), AIM2 (66902-1-Ig, Proteintech), ASC (67494-1-Ig, Proteintech), ATP (ab76020, Abcam), Lamin B1 (66095-1-Ig, Proteintech), pH2A.X (9718, CST). Membranes were then washed and incubated with the appropriate horseradish peroxidase (HRP)-conjugated secondary antibodies (CoWin Biotech, anti-rabbit (CW0103) 1:10000, anti-mouse (CW0102) 1:10000. Proteins were visualized using Immobilon Forte Western HRP Substrate (Millipore, WBLUF0500).

### Immunofluorescence staining

In brief, cells were fixed in 4% paraformaldehyde (Yongjinbio) for 10 min and permeabilized with PBST containing 0.5% Triton X-100 for 10 min. Cells were then incubated in PBST containing 1% BSA for 30 min. Cells were incubated in primary antibodies (cleaved N-terminal GSDMD (ab215203, Abcam), cleaved N-terminal GSDME (ab222408, Abcam), pMLKL (ab187091, Abcam) overnight at 4 °C in PBST with 1% BSA. Cells were washed three times in PBST, and then incubated in the appropriate secondary antibodies (Abcam) for 1 h at room temperature. Cells were washed three times in PBST. Cells were counterstained with DAPI (S2110, Solarbio). Images were acquired by confocal laser scanning microscopy (LSM780; Carl Zeiss) using a 40× Apochromat objective.

### Multiplex immunohistochemistry and image acquisition

The histological analysis was performed on formalin-fixed paraffin-embedded (FFPE) mouse tumors. FFPE tissue sections were deparaffinized by xylene and rehydrated by gradient alcohol. After an initial deparaffinization procedure, a heat-induced epitope retrieval (HIER) in EDTA buffer (pH 9.0) at 100 °C for 30 min was performed on all slides. Subsequently, several rounds of staining consisting of antigen retrieval, blocking, primary antibody, HRP-coupled secondary antibody, and tyramide signal amplification (TSA) steps were performed. The following primary antibodies were used: (cleaved N-terminal GSDMD (36425, CST), cleaved N-terminal GSDME (ab222407, Abcam), pMLKL (37333, CST), Perforin (A0093, ABclonal), Granzyme B (46890, CST), F4/80 (70076, CST), CD8α (98941, CST), NK1.1 (ab289542, Abcam), TNF-α (60291-1-Ig, Proteintech), IFN-γ (ET1703-17, Huabio), Cleaved Caspase-3 (9664, CST), Cleaved Caspase-8 (9664, CST). To visualize immunofluorescent signaling, PANO 7-plex IHC kit (0004100100, Panovue) was used: PPD480, PPD520, PPD570, PPD650, PPD780. DAPI (Panovue) was used for the nuclear counterstain. Imaging of slides was performed on SLIDEVIEW VS200 (Olympus). First, an overview of the whole tissue (20x magnification) was scanned. Then High-resolution images of intratumoral and marginal tumor regions were analyzed by the VS200 ASW Software.

### Animal studies

All animal procedures were performed in accordance with national and institutional guidelines from the Institutional Animal Care and Use Committees and approved by the Ethical Commission of Sun Yat-Sen University. The number of mice and the inclusion/exclusion criteria were based on institutional guidelines. 6–8-week-old female BALB/c mice were obtained from the Laboratory Animal Research Center of South China University of Technology. A minimum of five mice per group were used in all experiments. We measured tumor size in accordance with institutional guidelines. Tumor size was measured twice every week, and calculated using the formula: V= (Width2×Length)/2 and reported as tumor mass volume (mm^3^). The experiments were not randomized and the investigators were not blinded.

### Statistical analyses

Statistical analyses were performed using GraphPad Prism software. The method of statistical analysis was indicated in the figure legend. For cell culture experiments, statistical differences were calculated using a two-tailed unpaired Student’s *t*-test. One-way ANOVA was used for statistical significance in tumor growth. All data are presented as mean ± s.e.m., a value of *P* < 0.05 was considered statistically significant (*P < 0.05; **P < 0.01; ***P < 0.001; ****P < 0.0001; NS, not significant).

### Supplementary information

Supplemental Material

## Data Availability

All data generated or analyzed during this study are included in this paper. Supplementary Information is available in the online version of the paper.
