# Supplementary material for: Tumors cells with mismatch repair deficiency induce hyperactivation of pyroptosis resistant to cell membrane damage but are more sensitive to co-treatment of IFN-γ and TNF-α to PANoptosis
Source: Cell Death Discov. 2024 May 13;10:227. doi: 10.1038/s41420-024-01984-7 (PMC11091123; doi:10.1038/s41420-024-01984-7)
Supplement: Supplementary file 1 — Supplemental Material [file 41420_2024_1984_MOESM1_ESM.pdf]

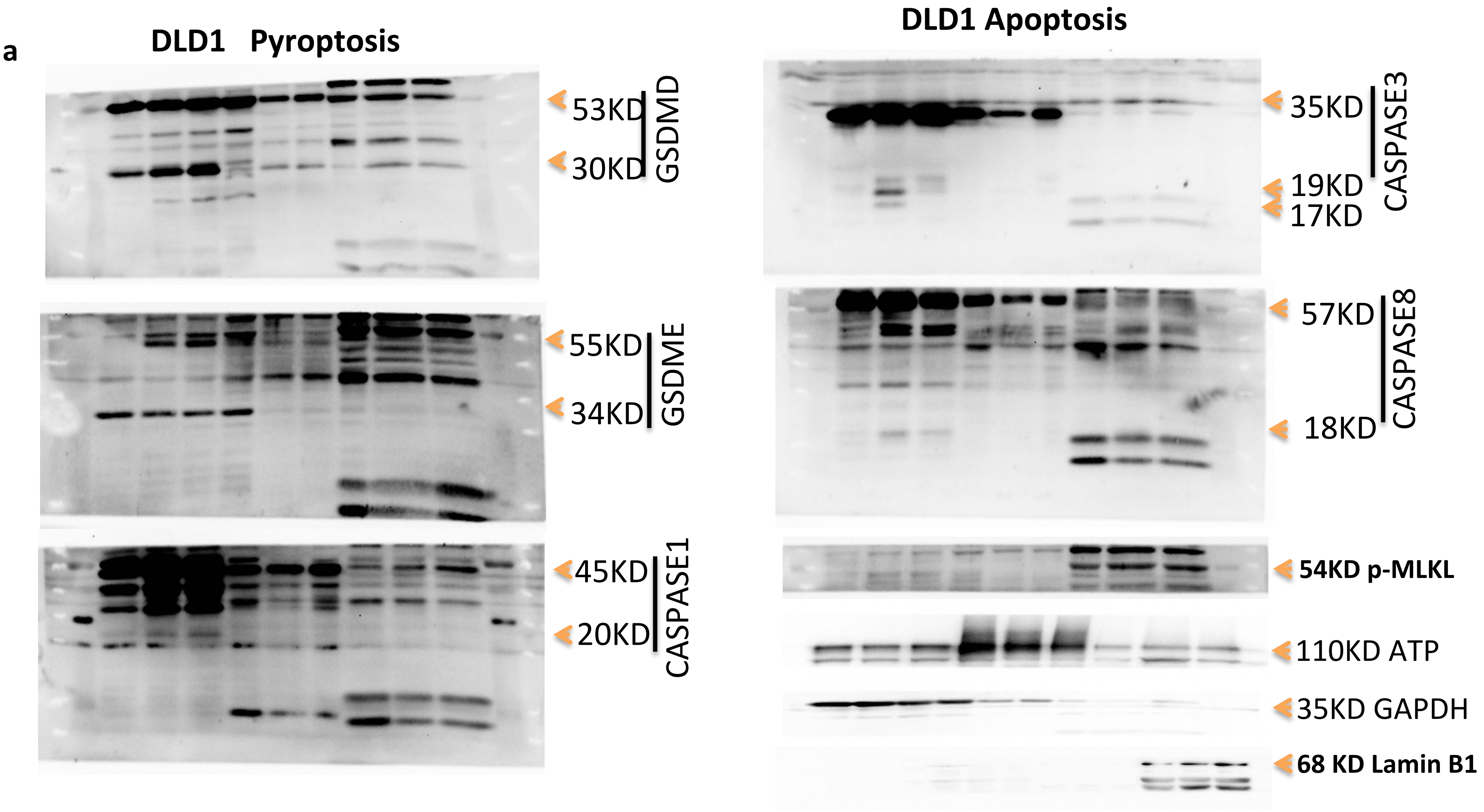

Fig.4 | The effect of inhibitors on PANoptosis induced by co-treatment of IFN-γ and TNF-α.

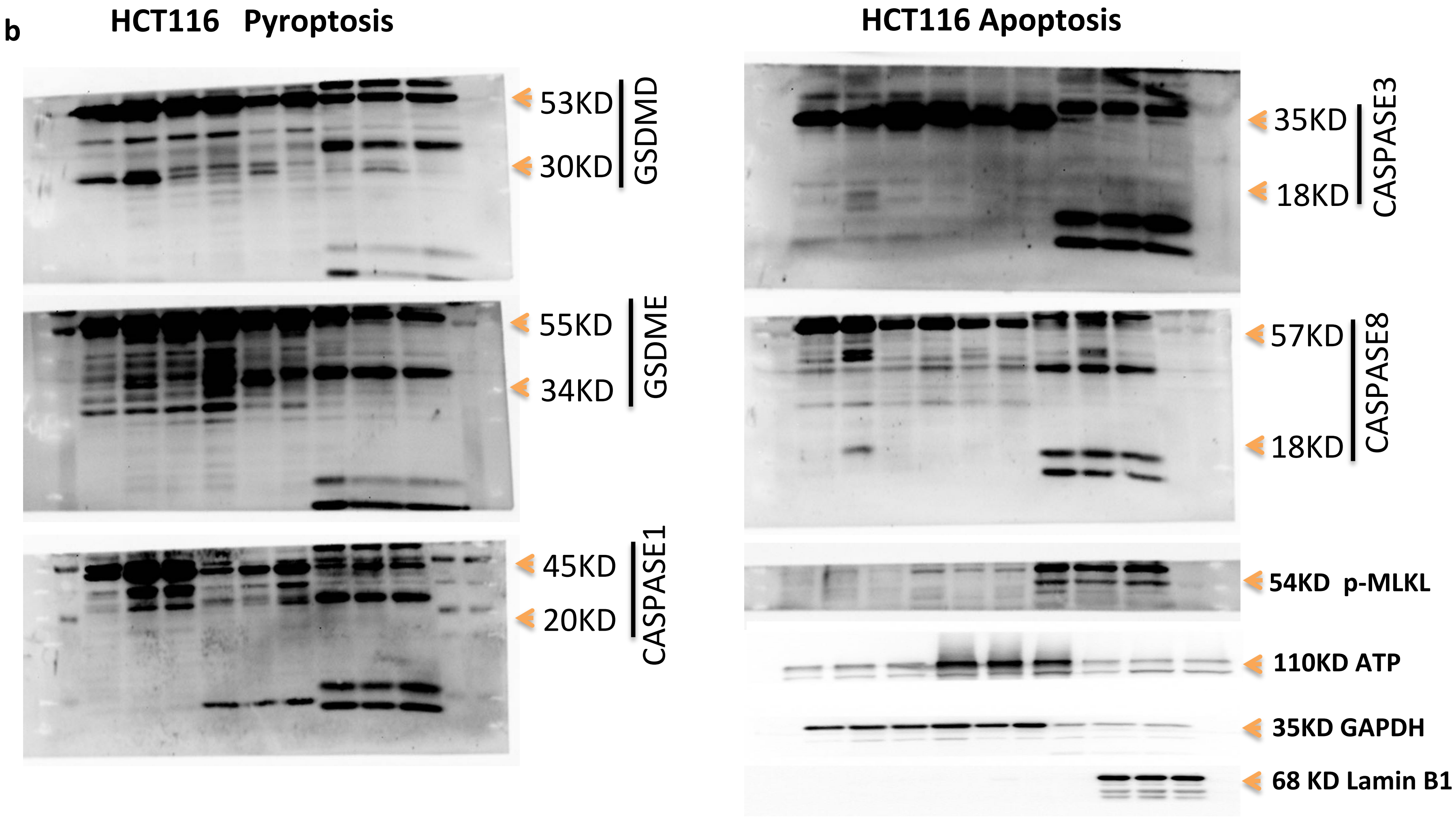

Fig.4 | The effect of inhibitors on PANoptosis induced by co-treatment of IFN-γ and TNF-α.

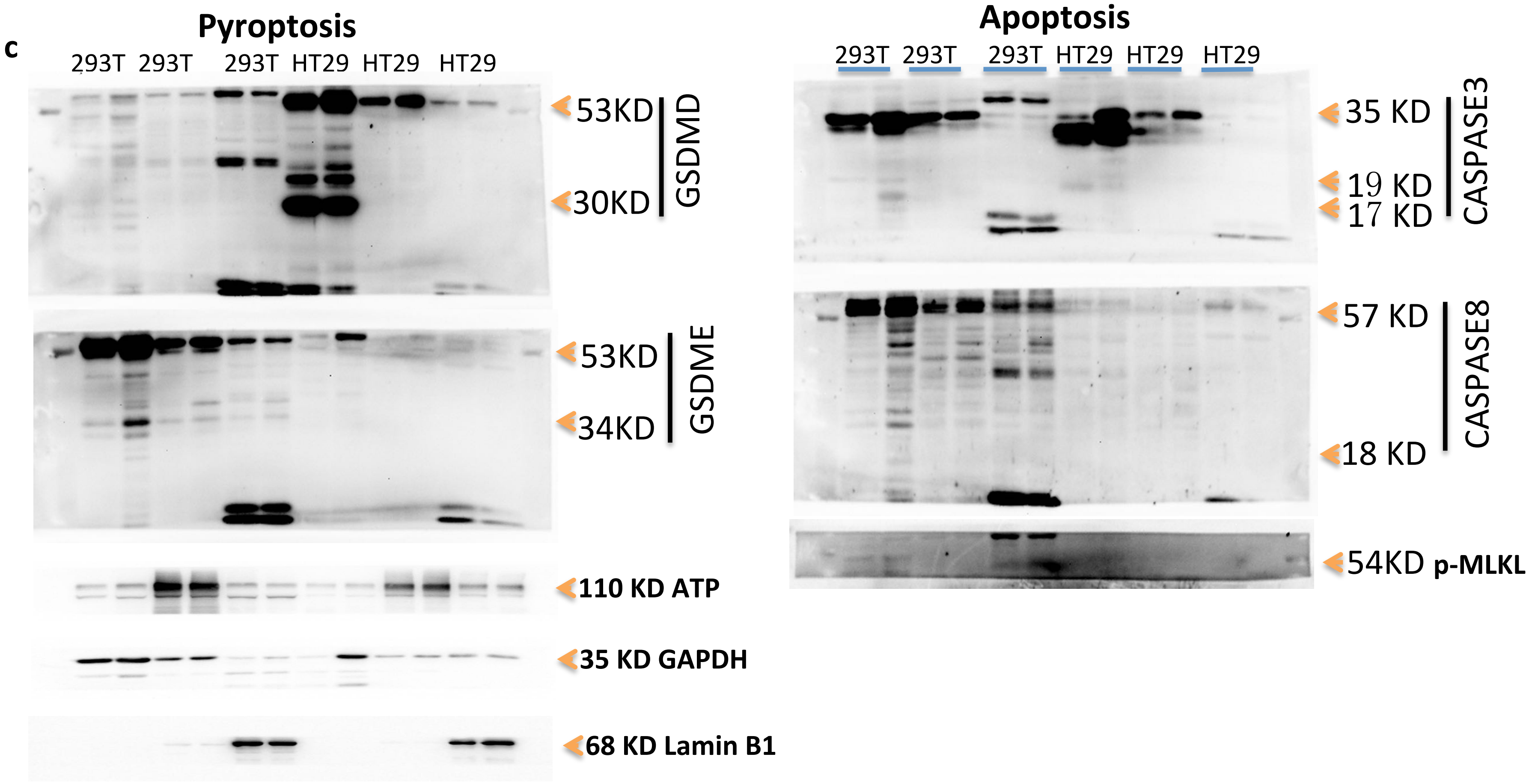

Fig.4 | The effect of inhibitors on PANoptosis induced by co-treatment of IFN-γ and TNF-α.

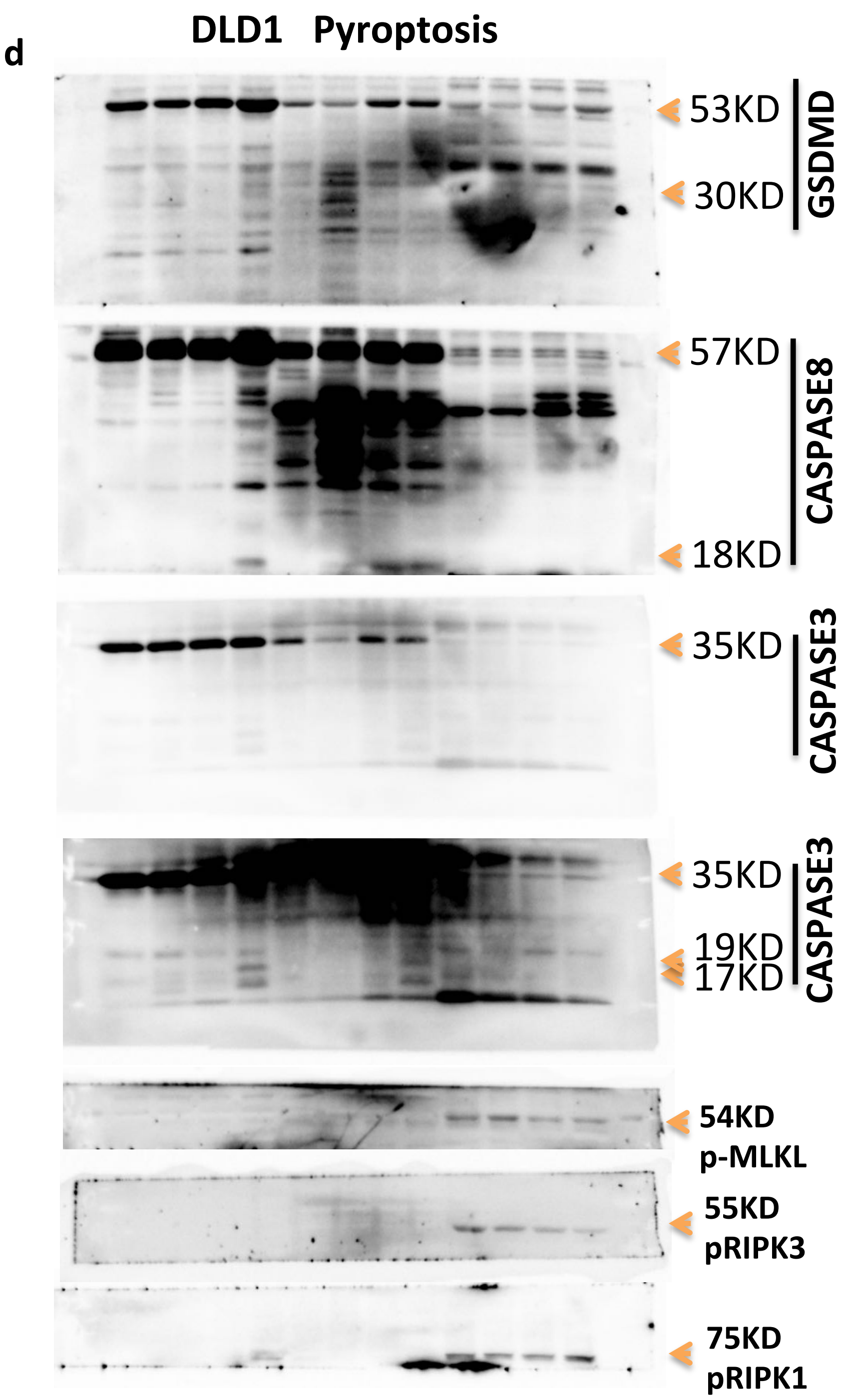

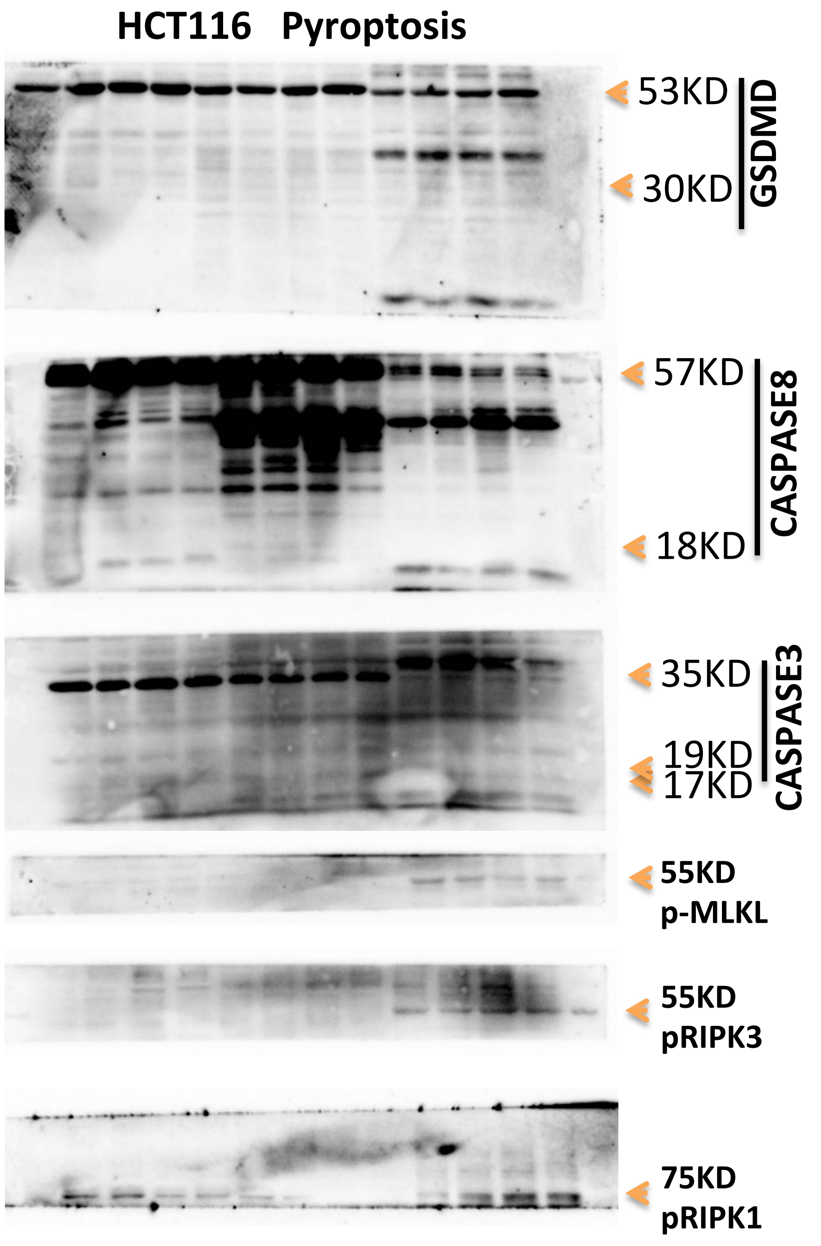

Fig.4 | The effect of inhibitors on PANoptosis induced by co-treatment of IFN-γ and TNF-α.

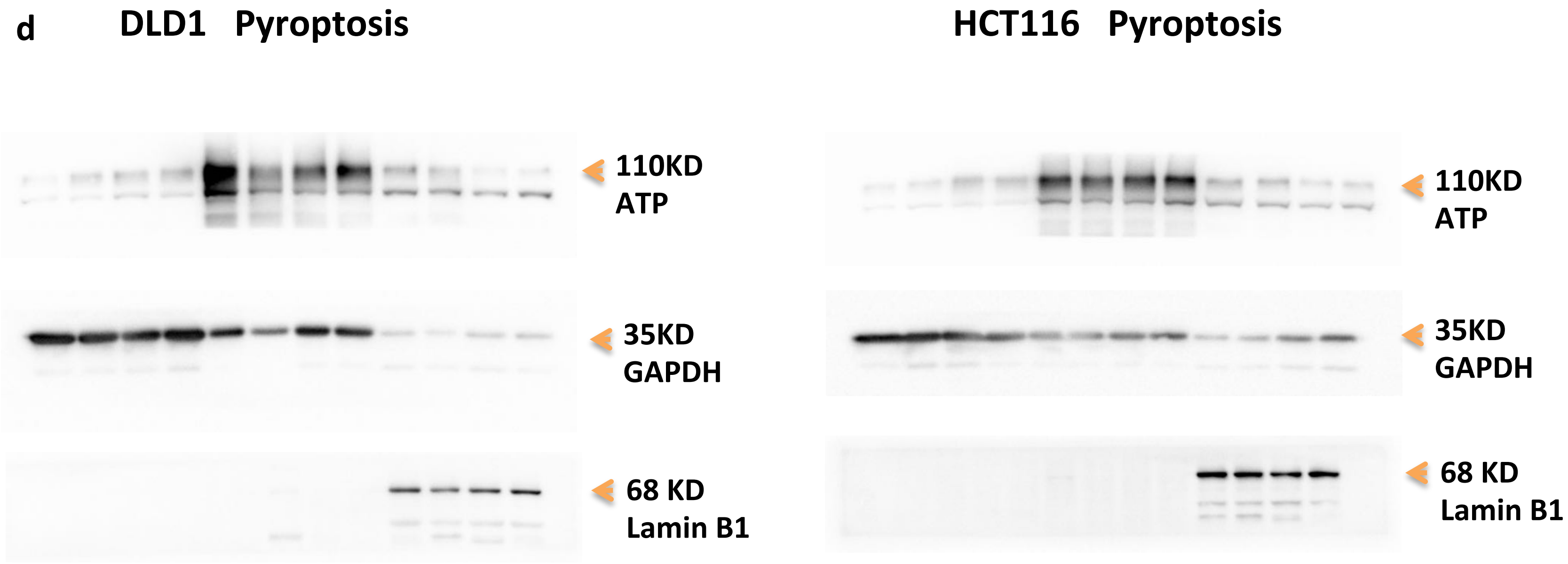

Fig.4 | The effect of inhibitors on PANoptosis induced by co-treatment of IFN-γ and TNF-α.

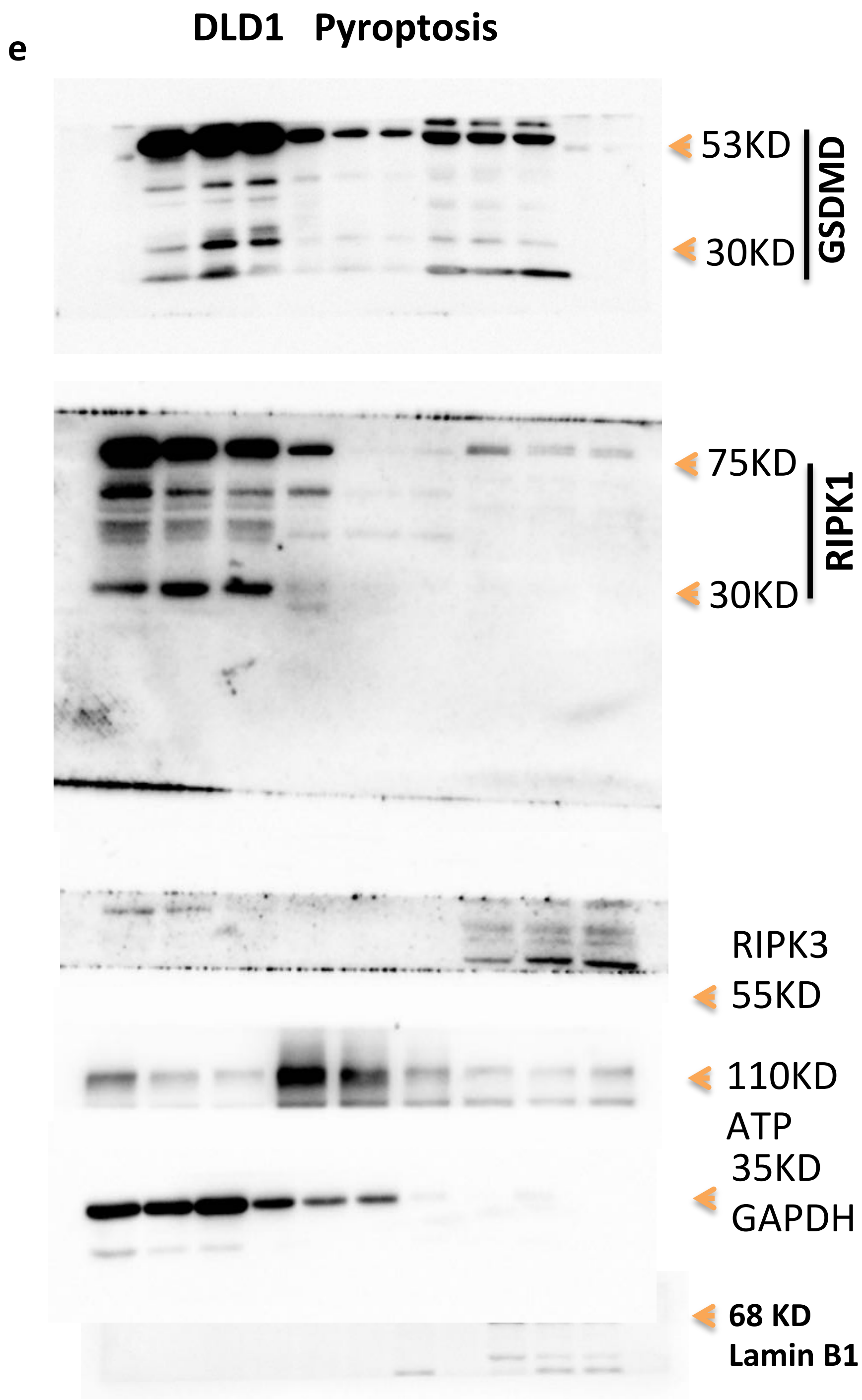

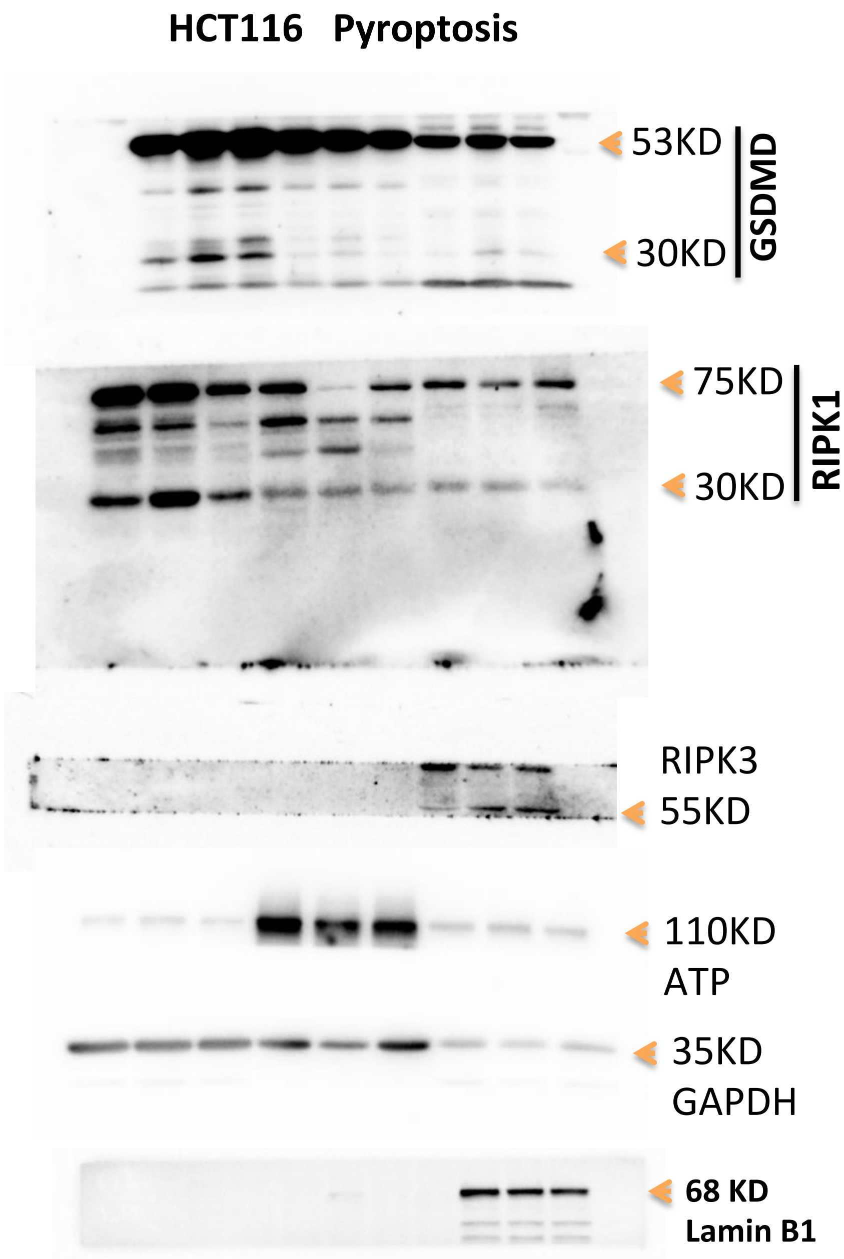

Fig.4 | The effect of inhibitors on PANoptosis induced by co-treatment of IFN-γ and TNF-α.

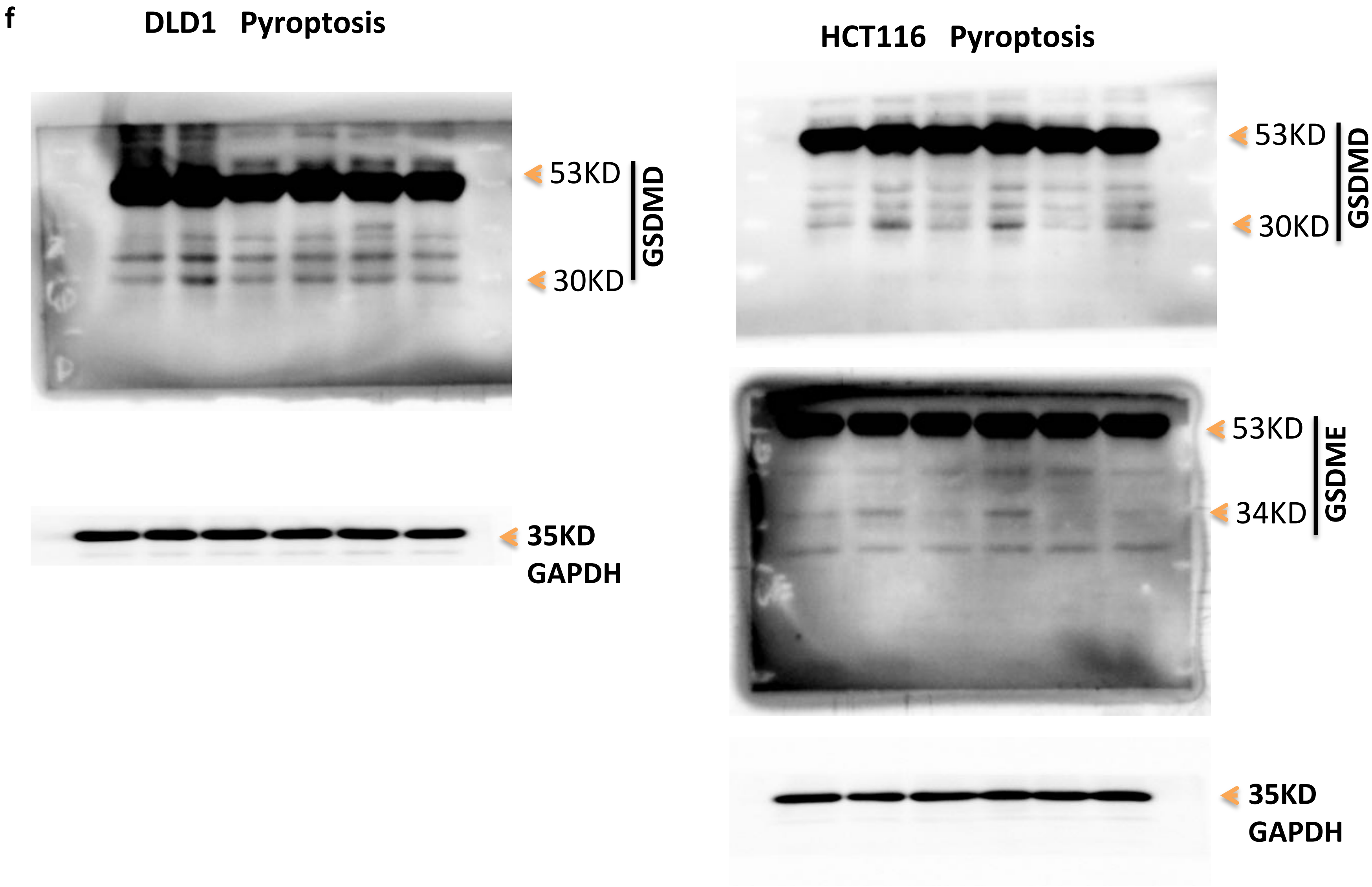

Fig.4 | The effect of inhibitors on PANoptosis induced by co-treatment of IFN-γ and TNF-α.

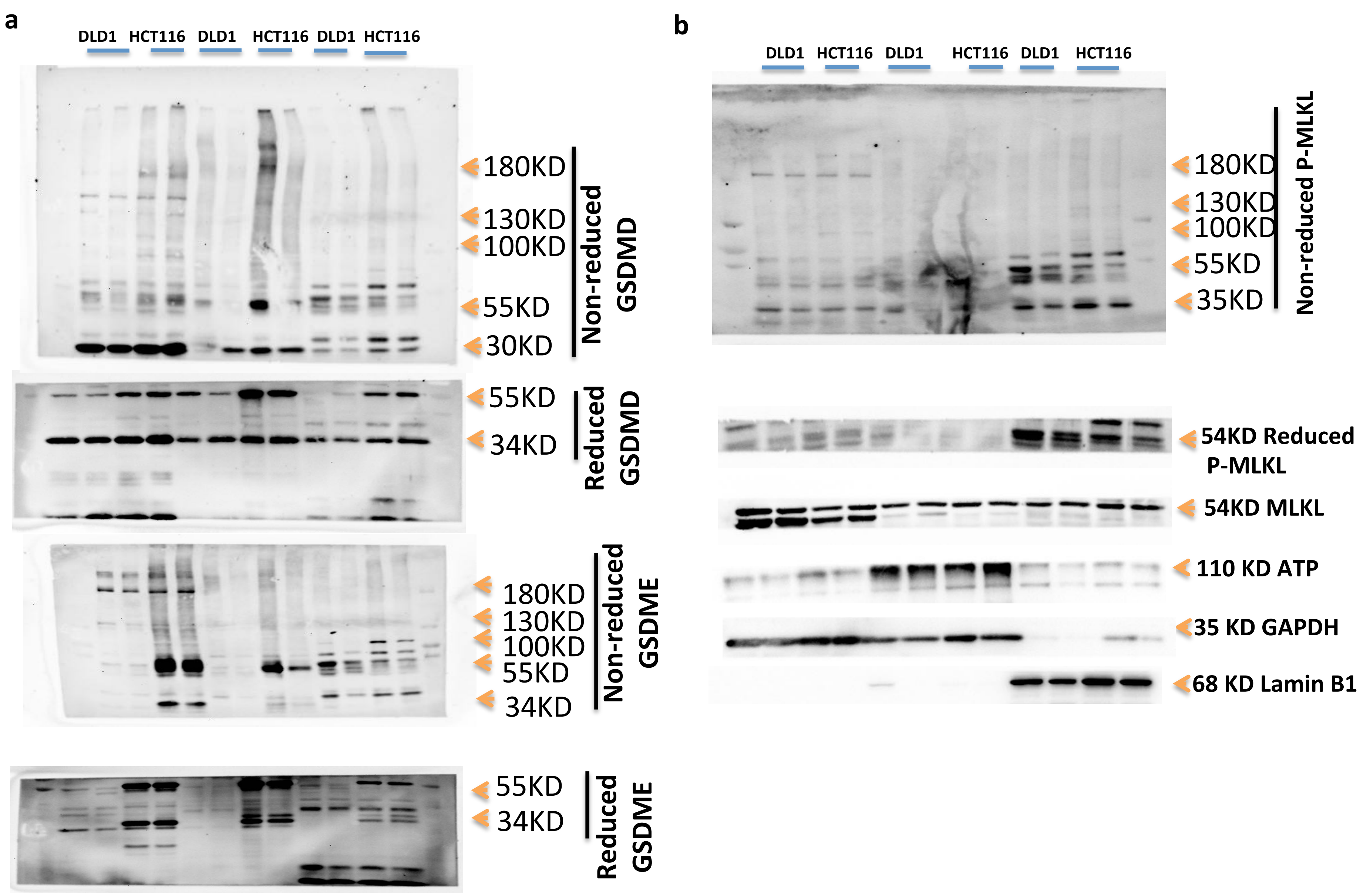

Fig.5 | Oligomerization of NT-GSDMD, NT-GSDME and p-MLKL triggers PANoptosis after co-treatment of IFN-γ + TNF-α.

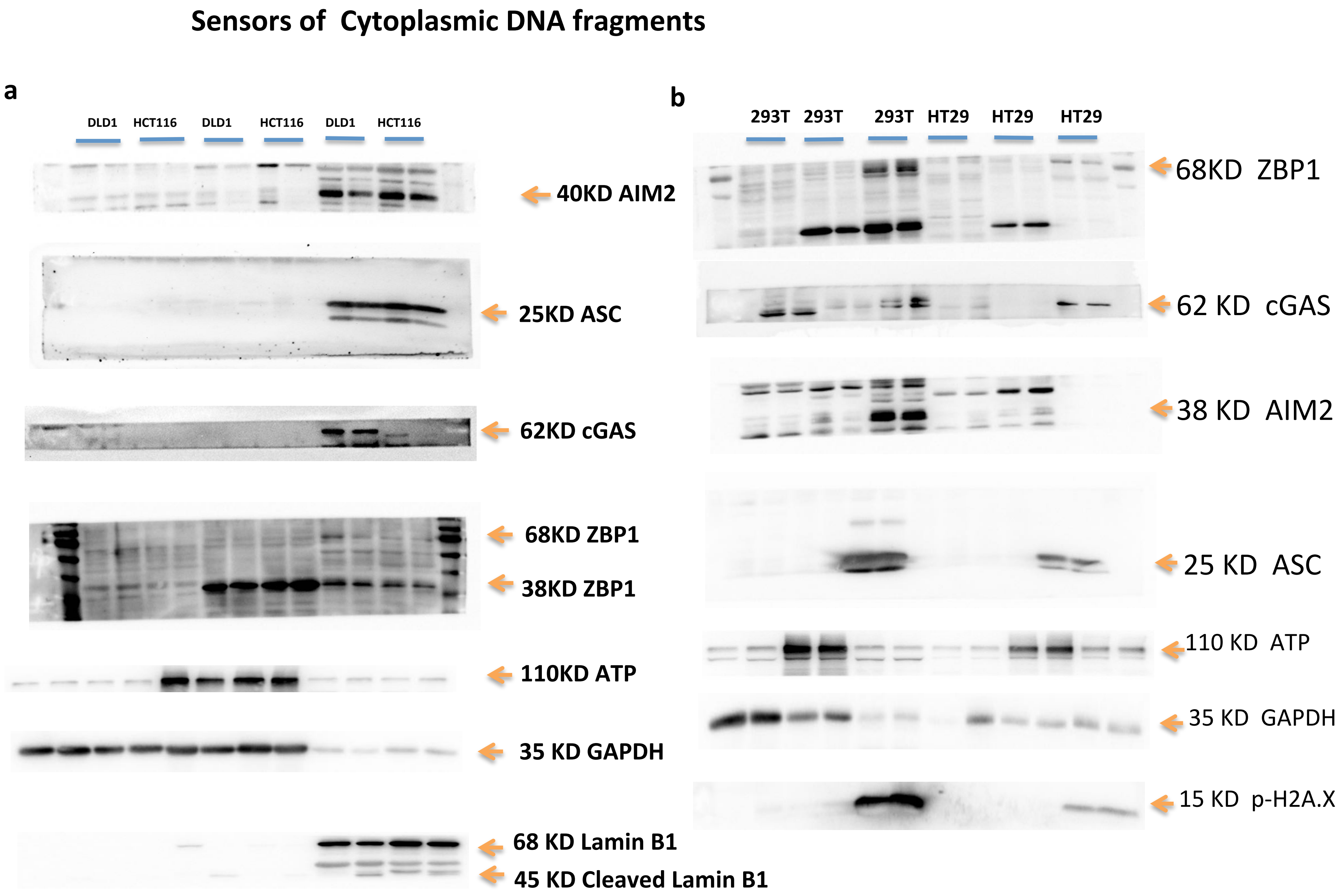

Fig.6 | DNA sensors for DNA fragments in Cancer cells with dMMR are in the nucleus, not in the cytoplasm.

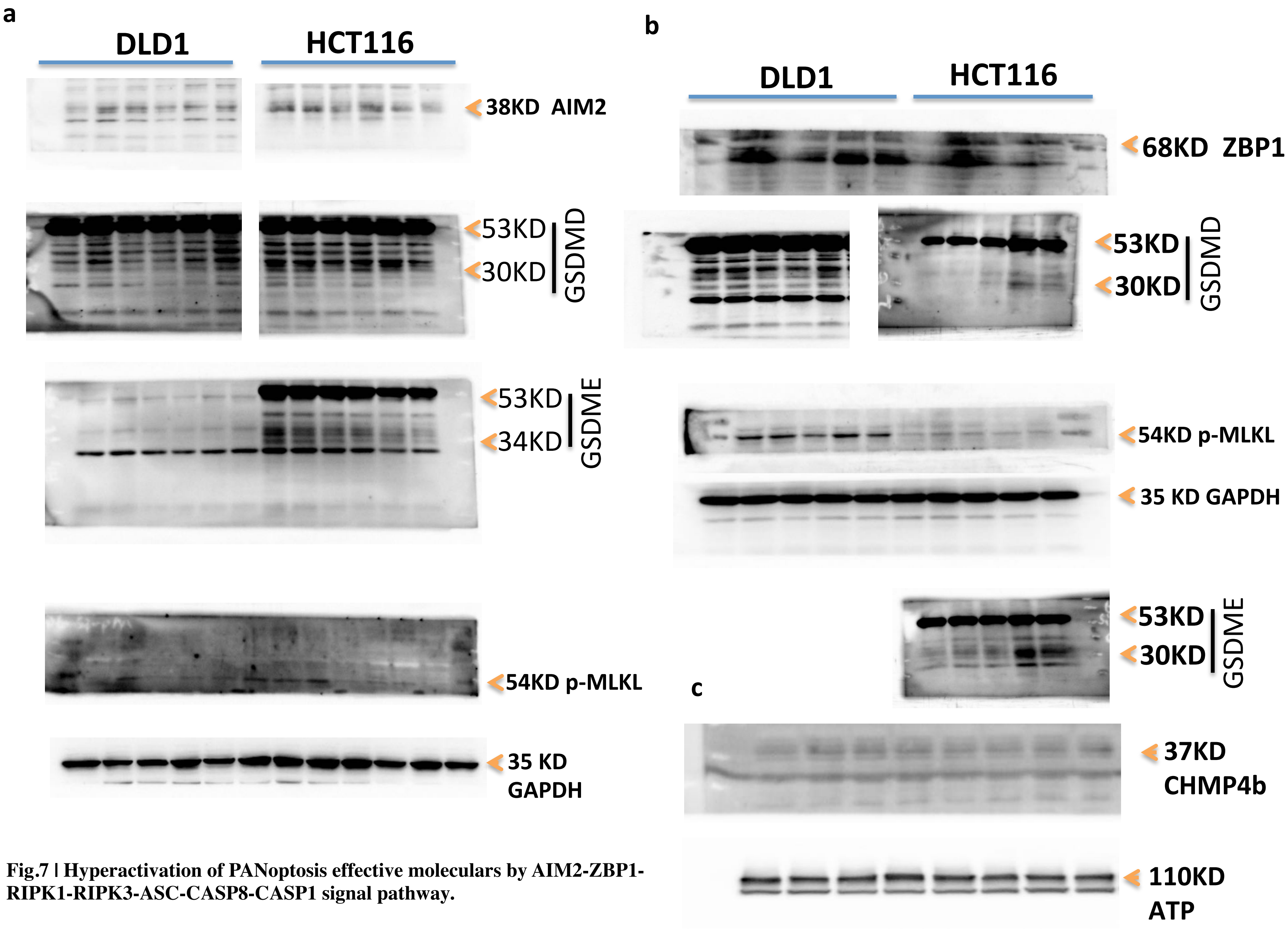

Fig.7 | Hyperactivation of PANoptosis effective moleculars by AIM2-ZBP1-RIPK1-RIPK3-ASC-CASP8-CASP1 signal pathway.

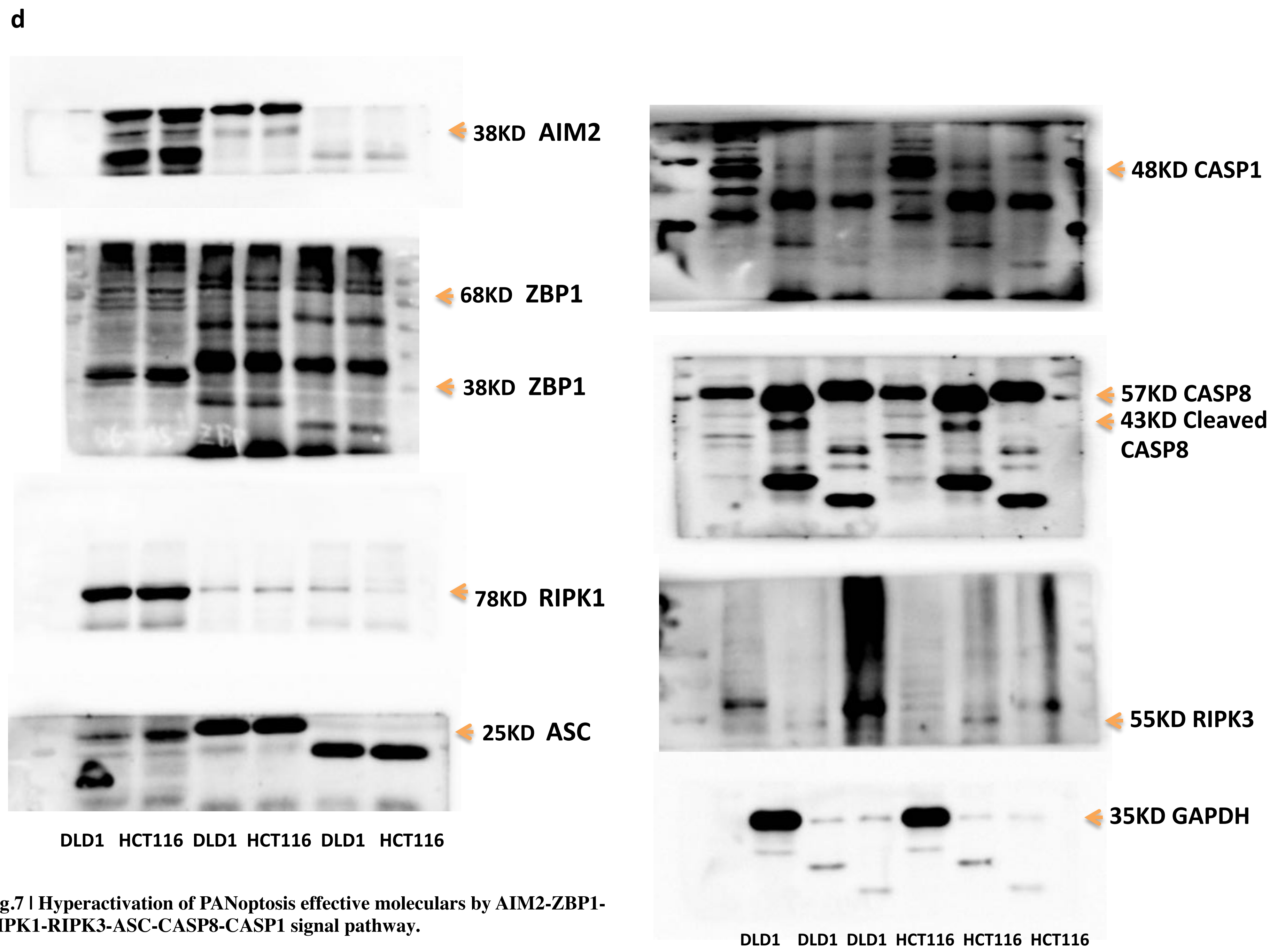

Fig.7 | Hyperactivation of PANoptosis effective moleculars by AIM2-ZBP1-RIPK1-RIPK3-ASC-CASP8-CASP1 signal pathway.

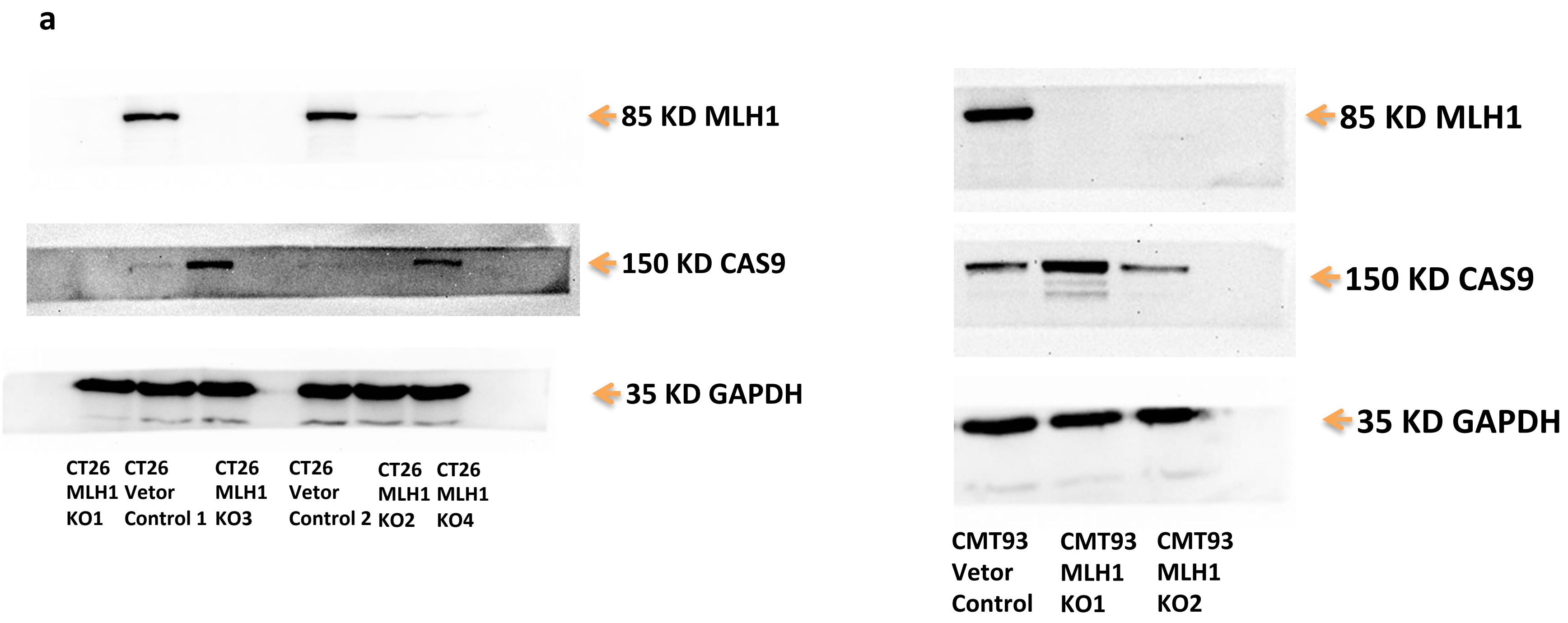

Fig.10 | *Mlh1* knockout CT26 and CMT93 undergo natural hyperactivation of GSDMD, GSDME and p-MLKL.

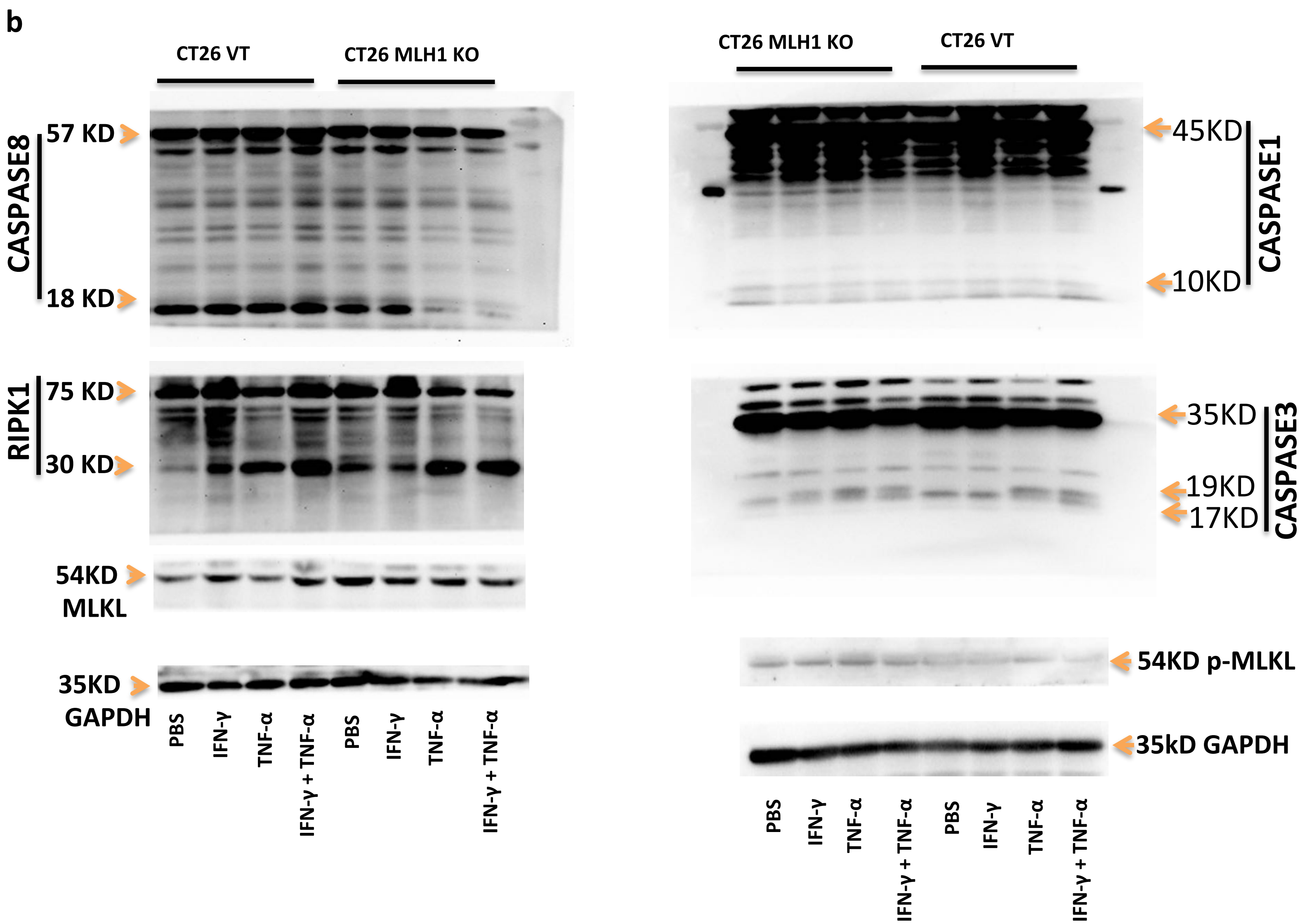

Fig.10 | *Mlh1* knockout CT26 and CMT93 undergo natural hyperactivation of GSDMD, GSDME and p-MLKL.

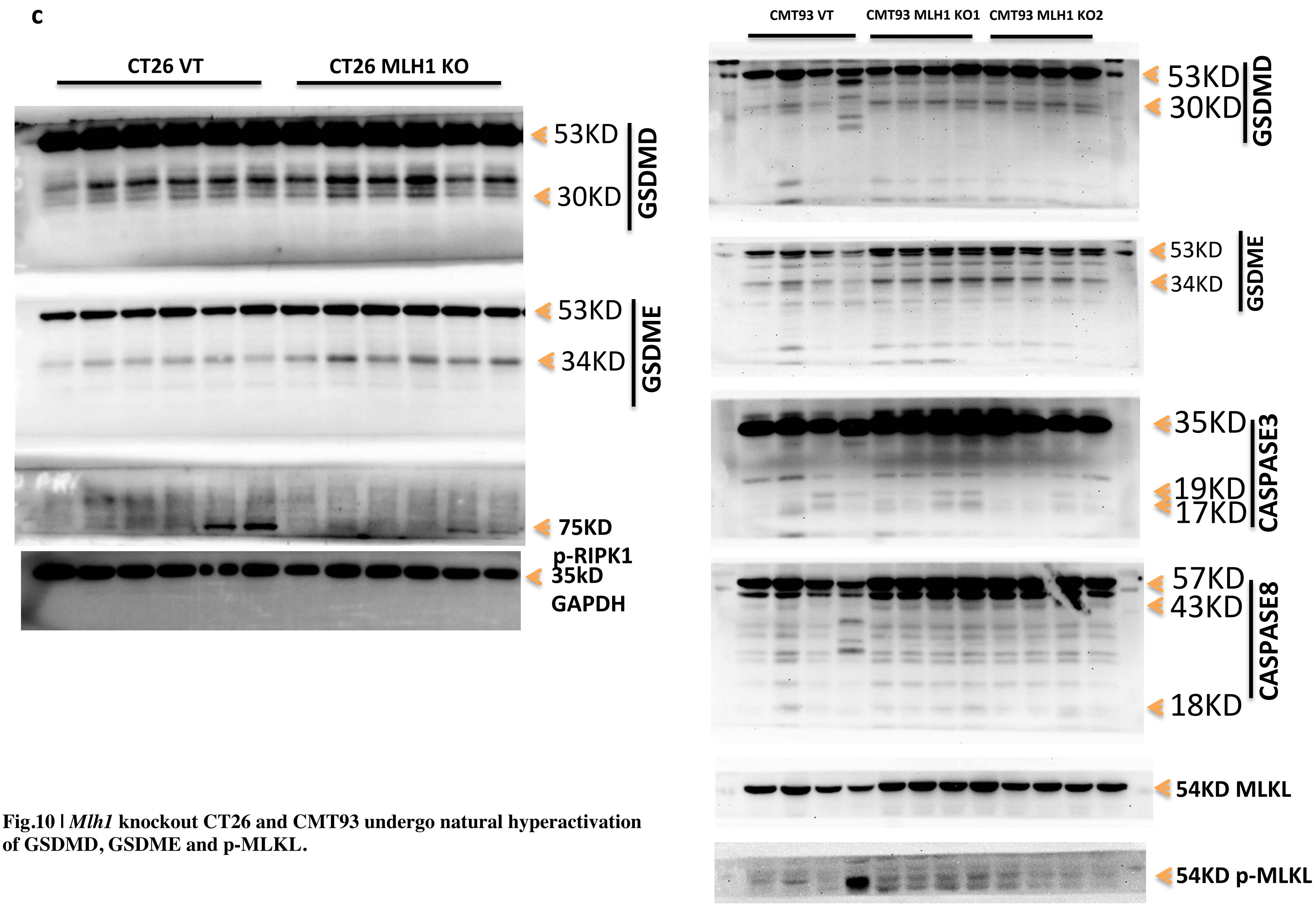

Fig.10 | *Mlh1* knockout CT26 and CMT93 undergo natural hyperactivation of GSDMD, GSDME and p-MLKL.

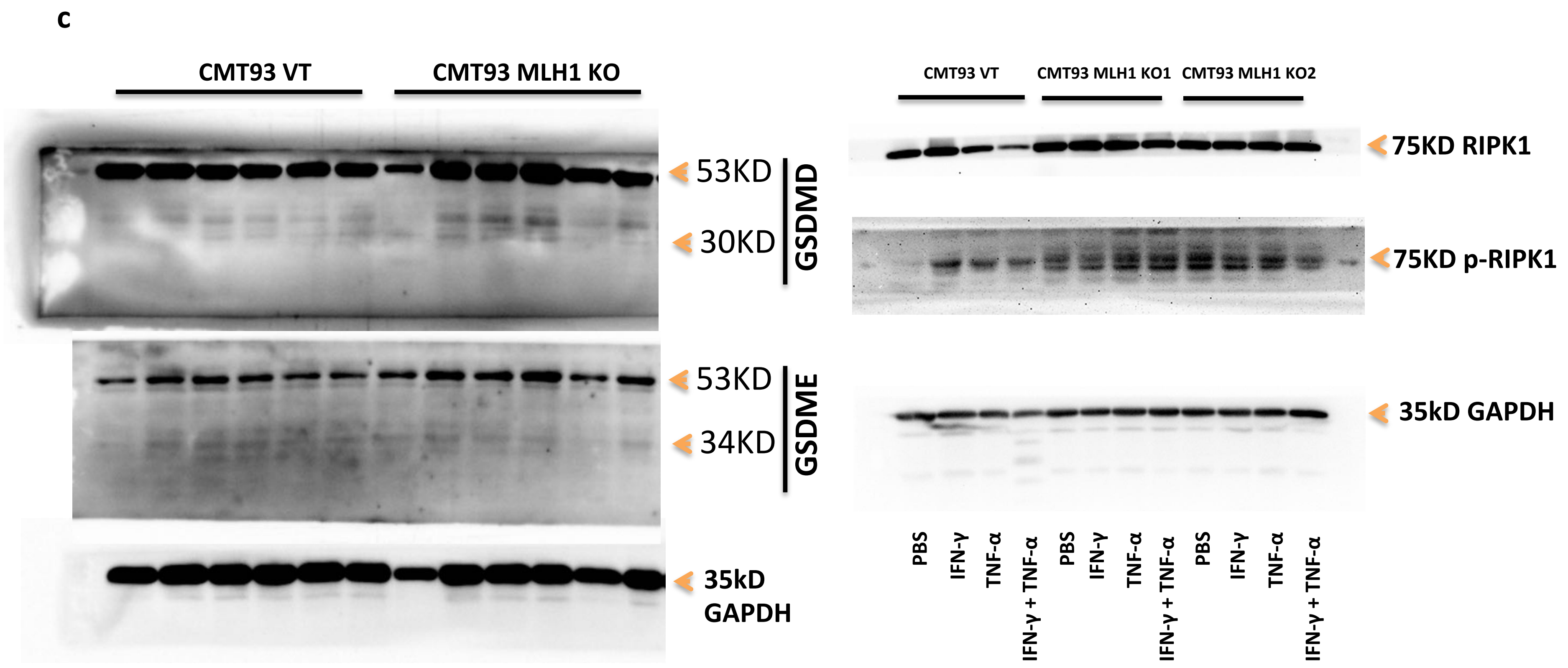

Fig.10 | *Mlh1* knockout CT26 and CMT93 undergo natural hyperactivation of GSDMD, GSDME and p-MLKL.

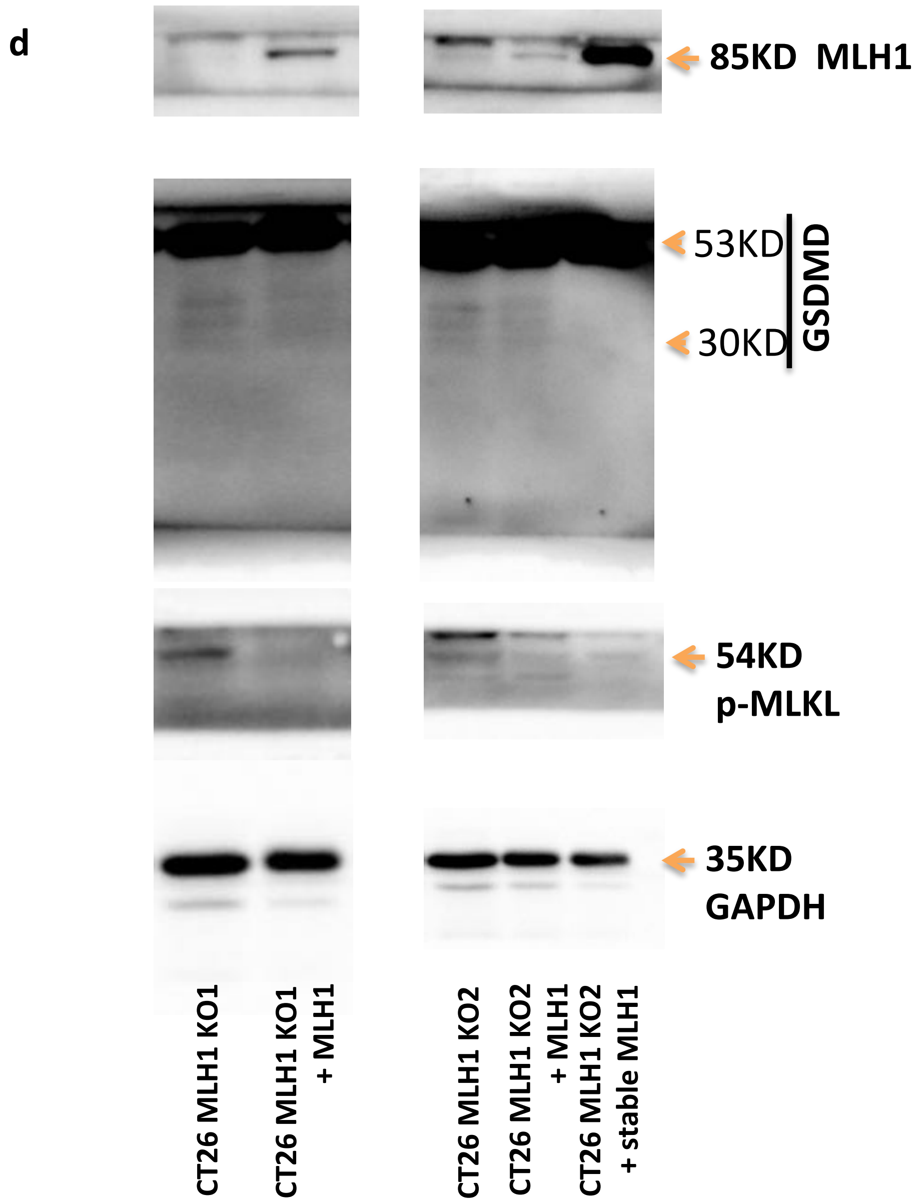

Fig.10 | *Mlh1* knockout CT26 and CMT93 undergo natural hyperactivation of GSDMD, GSDME and p-MLKL.
